# Comprehensive Risk Analysis of Emergency Medical Response Systems in Serbian Healthcare: Assessing Systemic Vulnerabilities in Disaster Preparedness and Response

**DOI:** 10.3390/healthcare12191962

**Published:** 2024-10-01

**Authors:** Vladimir M. Cvetković, Jasmina Tanasić, Renate Renner, Vanja Rokvić, Hatiža Beriša

**Affiliations:** 1 Department of Disaster Management and Environmental Security, Faculty of Security Studies, University of Belgrade, Gospodara Vučića 50, 11040 Belgrade, Serbia; vanjarokvic@fb.bg.ac.rs; 2 Scientific-Professional Society for Disaster Risk Management, Dimitrija Tucovića 121, 11040 Belgrade, Serbia; 3 International Institute for Disaster Research, Dimitrija Tucovića 121, 11040 Belgrade, Serbia; 4 Safety and Disaster Studies, Department of Environmental and Energy Process Engineering, Montanuniversität of Leoben, Franz Josef-Straße 18, 8700 Leoben, Austria; renate.renner@unileoben.ac.at; 5 Standing Conference of Towns and Municipalities, Makedonska 22/VIII, 11103 Belgrade, Serbia; jasmina.tanasic@skgo.org; 6 Military Academy, University of Defence, Veljka Lukića Kurjaka, 11042 Belgrade, Serbia; hatidza.berisa@mod.gov.rs

**Keywords:** risk, disaster, emergency preparedness, effectiveness, comprehensive survey, emergency medical response systems, vulnerabilities, Serbian healthcare

## Abstract

Background/Objectives: Emergency Medical Response Systems (EMRSs) play a vital role in delivering medical aid during natural and man-made disasters. This quantitative research delves into the analysis of risk and effectiveness within Serbia’s Emergency Medical Services (EMS), with a special emphasis on how work organization, resource distribution, and preparedness for mass casualty events contribute to overall disaster preparedness. Methods: The study was conducted using a questionnaire consisting of 7 sections and a total of 88 variables, distributed to and collected from 172 healthcare institutions (Public Health Centers and Hospitals). Statistical methods, including Pearson’s correlation, multivariate regression analysis, and chi-square tests, were rigorously applied to analyze and interpret the data. Results: The results from the multivariate regression analysis revealed that the organization of working hours (*β* = 0.035) and shift work (*β* = 0.042) were significant predictors of EMS organization, explaining 1.9% of the variance (*R*^2^ = 0.019). Furthermore, shift work (*β* = −0.045) and working hours (*β* = −0.037) accounted for 2.0% of the variance in the number of EMS points performed (*R*^2^ = 0.020). Also, the availability of ambulance vehicles (*β* = 0.075) and financial resources (*β* = 0.033) explained 4.1% of the variance in mass casualty preparedness (*R*^2^ = 0.041). When it comes to service area coverage, the regression results suggest that none of the predictors were statistically significant. Based on Pearson’s correlation results, there is a statistically significant correlation between the EMS organization and several key variables such as the number of EMS doctors (*p* = 0.000), emergency medicine specialists (*p* = 0.000), etc. Moreover, the Chi-square test results reveal statistically significant correlations between EMS organization and how EMS activities are conducted (*p* = 0.001), the number of activity locations (*p* = 0.005), and the structure of working hours (*p* = 0.001). Conclusions: Additionally, the results underscore the necessity for increased financial support, standardized protocols, and enhanced intersectoral collaboration to strengthen Serbia’s EMRS and improve overall disaster response effectiveness. Based on these findings, a clear roadmap is provided for policymakers, healthcare administrators, and EMS personnel to prioritize strategic interventions and build a robust emergency medical response system.

## 1. Introduction

Among various healthcare services, Emergency Medical Response Systems (EMRS) (Appendix B) are an important element serving for urgent needs of disasters, which refer to both natural hazards and man-made (technological disasters) [1,2,3]. Considering that, the effectiveness of these systems can have a profound impact on patient outcomes, especially in critical, life-threatening situations [4,5]. Also, it is important to mention that, in Serbia, the healthcare sector has encountered numerous obstacles, such as economic limitations, resource scarcity, and ongoing reform transitions, which influence the performance of its EMRS [6,7,8,9]. Following that, the evolution of emergency medical services (EMS) in Serbia has progressed alongside the country’s broader healthcare system, reflecting significant socio-economic and political shifts [10,11,12]. In that way, the EMS framework in Serbia has historically been shaped by the Yugoslavian healthcare model, which emphasized accessibility and comprehensiveness [13,14]. However, the disintegration of Yugoslavia and the ensuing conflicts in the 1990s caused substantial disruptions in healthcare delivery, including emergency services [14].

It is crucial to point out that reforms initiated post-2000 aimed to align Serbian EMS with European standards and have had varying degrees of success. Likewise, initial reform efforts concentrated on restructuring organizational frameworks, boosting funding, and enhancing training programs for medical personnel [15,16,17]. Despite these reforms, ongoing issues such as inadequate infrastructure and an insufficient workforce continue to challenge the system’s ability to deliver timely and effective emergency care [18]. In addition, Serbia’s EMRS is currently organized into several key components: pre-hospital care, hospital emergency departments, and specialized emergency units [12,19]. Pre-hospital care involves dispatch centres and ambulance services, which are crucial for ensuring rapid response times. According to the provisions of the Law on Healthcare (“Official Gazette of the Republic of Serbia”, No. 25 of 3 April 2019, 92 of 27 October 2023), one of the main priorities of healthcare workers and institutions at all levels of the healthcare system can be said to be the provision of EMS. In the treatment of emergency cases and conditions, the basic principles and methods of emergency medicine are applied). Furthermore, the organization of emergency medical services is based on two interconnected subsystems: pre-hospital emergency care and hospital-based emergency care. Pre-hospital emergency medical care is a continuous activity of primary healthcare institutions and includes providing medical assistance at the site of the emergency or within a healthcare institution, medical transport of critically ill or injured patients to hospital facilities, with continuous monitoring and provision of assistance during transport. This assistance is provided as part of the regular activities of doctors and their associates, as well as through the work of on-call or emergency teams during the night, weekends, and public holidays. On the other side, in health centres that serve territories with more than 25,000 inhabitants, an emergency medical service can be organized for the continuous reception and care of emergency cases. On the other hand, hospital-based emergency medical care is provided through the work of specialized teams in the emergency departments of general hospitals, clinical-hospital centers, clinics, institutes, and university clinical centers, with the admission of patients for hospital treatment. Healthcare institutions that are unable to adequately care for patients are required to organize medical transport and provide appropriate professional assistance during the transport to the most suitable hospital facility.

Nonetheless, research indicates that response times in Serbia frequently surpass international benchmarks due to logistical challenges and resource limitations [20]. One important aspect is that hospital emergency departments in Serbia grapple with issues such as overcrowding, understaffing, and outdated equipment [21]. These problems are exacerbated by the limited presence of specialized emergency units, like trauma and cardiac care centres, particularly in rural regions [1,4,8,12,15,21]. The effectiveness of EMRS in Serbia is further hampered by the uneven distribution of healthcare resources and disparities in access to emergency care between urban and rural populations [22,23,24]. Several risk factors impact the performance of EMRS in Serbia, including systemic, organizational, and operational challenges [25]. The Serbian healthcare system is plagued by chronic underfunding, affecting all levels of healthcare delivery, including emergency services [26]. Limited financial resources result in inadequate investment in infrastructure, technology, and human resources, leading to subpar EMRS performance [9,15,24,27]. An essential point to highlight is that operational risks include delays in response times, insufficient training for EMS personnel, and lack of access to advanced medical equipment. These factors contribute to increased morbidity and mortality rates among emergency patients [28]. Additionally, emergency medical technicians and paramedics often experience high levels of occupational stress and burnout, which can adversely affect their performance and decision-making abilities [6,29].

The effectiveness of EMRS in Serbia is assessed through various indicators, including response times, patient outcomes, and system efficiency. Response time is a crucial indicator of EMRS efficacy, as it directly influences patient survival rates, especially in cases of cardiac arrest, trauma, and stroke [30]. Studies reveal that response times in Serbia often exceed recommended international standards, mainly due to logistical challenges and resource constraints [6,31,32]. System efficiency is influenced by resource allocation, coordination among healthcare sectors, and the implementation of evidence-based protocols [33]. Efforts to improve system efficiency in Serbia have focused on enhancing communication systems, optimizing resource allocation, and implementing training programs for EMS personnel [1,2,21,34].

Addressing the risks and enhancing the efficacy of EMRS in Serbia requires a multifaceted approach involving policy, organizational, and operational interventions [35,36,37,38]. Organizational strategies should aim to enhance service delivery by improving communication systems, optimizing resource allocation, and implementing evidence-based protocols for emergency care [38]. Efforts to decentralize healthcare services and encourage regional collaboration can also help address disparities in access to emergency care [39]. Operational measures should concentrate on improving response times, enhancing training and education for EMS personnel, and ensuring access to advanced medical equipment [40].

This study thus analysed the risk spectrum and efficiency level in the Emergency Medical Services (EMS) of Serbia by exploring possible implications of work organization, distribution, and preparedness for major incidents on wider disaster readiness. Also, the primary goal of this study is to scientifically predict and explain the key factors influencing EMS performance while also identifying specific strategies and procedures that can improve the system’s efficiency during mass casualty incidents and other critical emergencies.

### Literature Review

It is important to mention that Emergency Medical Response Systems (EMRSs) worldwide have been extensively analyzed, providing valuable insights into their strengths, challenges, and best practices across various regions [5,6,33,38,41,42,43,44,45,46,47,48,49,50,51,52,53,54,55,56]. Conversely, an important observation is that studies from outside Serbia have identified common themes, such as the necessity for well-organized protocols [39,57], efficient resource distribution [43,58,59,60], and ongoing EMS personnel training [61,62]. Moreover, research from countries like Germany and the United Kingdom highlights the significance of standardized national protocols in ensuring consistent, high-quality care [10,63,64]. In Germany, where we have a high-quality system of standardized preclinical protocols (POLYQUALY), coordination of the emergency services has been improved and response times for all patients have decreased, as well as improved patient outcomes [65]. On the other side, the UK’s National Health Service (NHS) emphasizes standardized training and procedures, which have contributed to high survival rates in critical emergencies like cardiac arrests [66,67].

Effective resource allocation and infrastructure are key to EMRSs’ success [19,68]. A study in the US showed that urban ambulance services with advanced medical technology and well-equipped ambulances had better response times than their less-resourced rural counterparts [63,69,70,71]. Conversely, in countries with weaker infrastructure, longer response times have been linked to lower survival rates in trauma and cardiac emergencies [72]. Ongoing training is critical for effective EMS systems [70,73,74]. From the other perspective, Japan’s EMS training integrates disaster preparedness, which has proven effective in large-scale emergencies such as different disasters [75,76].

Technology has a crucial role in enhancing EMRS effectiveness [77,78]. So, advanced telemedicine systems allow EMS teams to provide real-time medical consultations, improving pre-hospital care quality [79]. Similarly, Scandinavian countries use GPS-based dispatch systems to optimize emergency vehicle deployment, reducing response times, particularly in rural areas [80]. In low–middle-income countries, resource limitations hinder EMRS efficiency [81]. However, community-based EMS models have shown promise in expanding access to care in remote areas [82]. Innovations like motorcycle ambulances have also helped overcome transportation challenges in urban areas with poor road infrastructure [83]. Furthermore, the presence of written mass casualty plans has been shown to significantly improve the performance of EMRS [42,71,84,85]. However, in Serbia, only 46% of institutions have dedicated emergency departments, and many lack specialized units for trauma and cardiac care [12].

In the context of post-conflict and post-disaster settings, Nelson et al. [7] discuss how health-system reforms are often complicated by unforeseen obstacles, frequently arising from inadequate initial evaluations. Expanding on this, Nelson et al. [12] conducted a comprehensive multimodal analysis of Serbia’s emergency medical services, blending both quantitative and qualitative methods. Comparing EMRS in Serbia with those in other countries provides insights into potential areas for improvement [50,63,64]. Conversely, Serbia faces challenges related to the lack of standardized protocols, limited access to advanced training, and insufficient funding [54,64]. These systemic weaknesses are reflected in Serbia’s Global Health Security Index (GHSI) rankings [86,87,88,89]. The World Bank’s Emergency Preparedness and Response Assessment [88] further illustrates the strain on Serbia’s medical response capabilities during disasters. The report highlights limited EMS capacity for advanced life support and the lack of prehospital mass casualty structures, including triage zones, medical costs, and transport staging areas [88,89,90].

## 2. Methods

**Figure 1:**
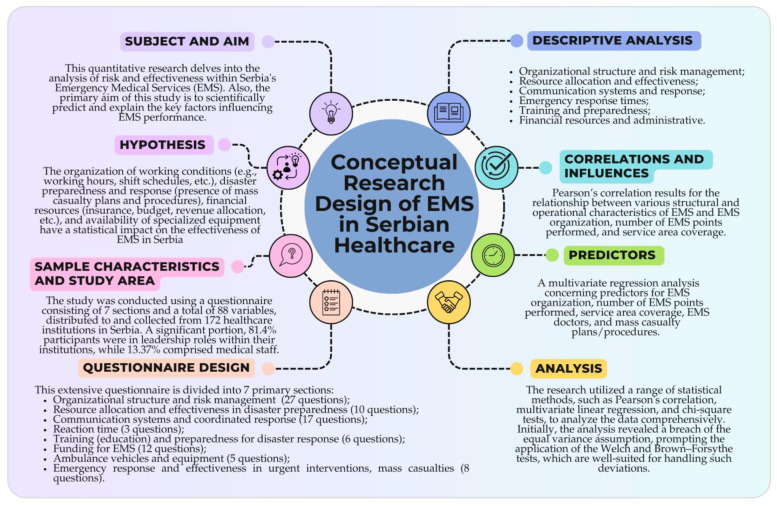
Conceptual research design of the emergency medical response system in Serbian healthcare.

This research delves into the analysis of risk and effectiveness within Serbia’s Emergency Medical Services (EMS), with a special emphasis on how work organization, resource distribution, and preparedness for mass casualty events contribute to overall disaster preparedness (see Figure 1). Also, the primary aim of this study is to scientifically predict and explain the key factors influencing EMS performance while also identifying specific strategies and procedures that can improve the system’s efficiency during mass casualty incidents and other critical disasters. Regarding that, this study utilizes quantitative methods, including Pearson’s correlation, multivariate regression analysis, and chi-square tests to identify key predictors of risk and efficacy in EMS performance. Additionally, it assesses how mass casualty plans and procedures impact the overall effectiveness of EMS, particularly during large-scale emergencies. The research was conducted from 2019/20 to 2022/23 in the areas of the mentioned local governments.

General Hypothesis—The organization of working conditions (e.g., working hours, shift schedules, etc.), disaster preparedness and response (presence of mass casualty plans and procedures), financial resources (insurance, budget, revenue allocation, etc.), and availability of specialized equipment have a statistical impact on the effectiveness of EMS in Serbia. Specific hypotheses are:

**H1.**  *The organization of working hours and shift schedules significantly improves EMS organization and performance in Serbia.*

**H2.**  *Financial resources* *allocated to EMS play a critical role in enhancing the system’s preparedness and operational efficiency during disaster response.*

**H3.**  *The* *implementation* *of mass casualty plans and procedures significantly strengthens EMS readiness and response capabilities in large-scale emergencies.*

**H4.**  *The availability of ambulance vehicles and specialized equipment significantly enhances the overall effectiveness of EMS, particularly in handling mass casualty incidents.*

### 2.1. Study Area

**Figure 2:**
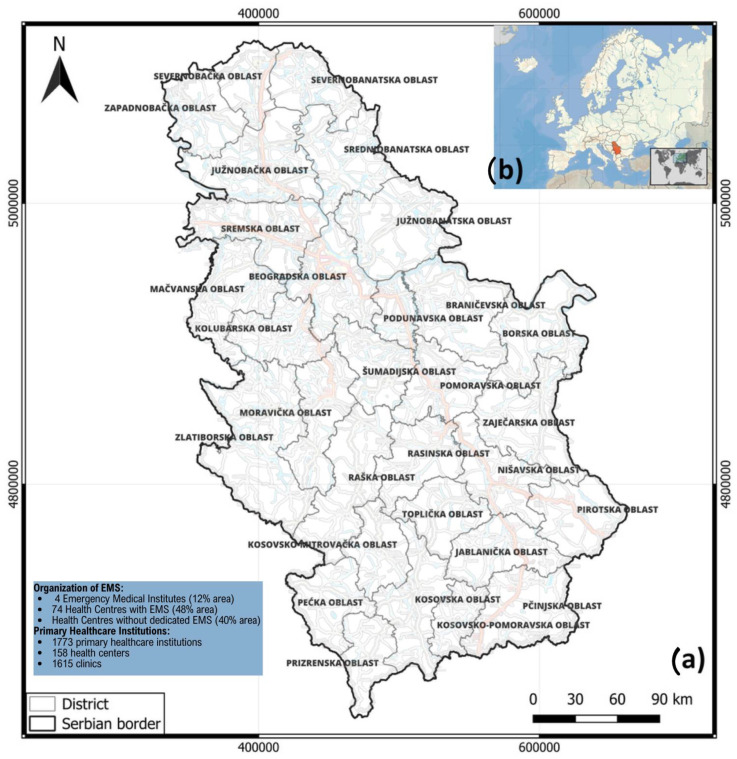
Map of Serbia showing districts and national borders. Subfigure (**a**) shows the scale in kilometers, indicating distances ranging from 0 to 90 km, while subfigure (**b**) illustrates Serbia’s location within Europe, providing a broader geographic context.

This research delves into evaluating emergency medical response systems within Serbia’s healthcare sector. In Southeast Europe, Serbia occupies the heart of the Balkan Peninsula (see Figure 2). The country, home to around 7 million inhabitants, features a varied landscape that includes fertile plains in the north and mountainous terrain in the south, each posing distinct challenges to emergency medical services (EMS) [1,2]. Serbia’s healthcare system is a hybrid model that combines state-run public healthcare with private medical services. Public healthcare operates on three levels: primary, secondary, and tertiary care. Emergency medical services, essential to this system, are primarily handled by the public sector. These services are designed to deliver urgent care both before patients reach the hospital and once they are within the hospital setting. The effectiveness and promptness of these services become especially crucial during disasters, whether natural, such as floods or earthquakes, or human-made [6,13,16,17,25,30,38].

According to the Statistical Office of the Republic of Serbia (https://www.stat.gov.rs/, accessed on 24 September 2024), based on the 2022 population census, approximately 6.7 million people live in Serbia. It is important to note that the birth rate is around 9.2 per 1000 inhabitants, while, in contrast, the mortality rate is 14.6 per 1000 inhabitants. These figures clearly indicate a negative natural population growth of about −5.4. Additionally, the average life expectancy in Serbia is 74.2 years (71.6 years for men and 77.3 years for women). Around 56% of the population lives in urban areas, with Belgrade being the most populous city. In terms of ethnic composition, Serbs make up about 83% of the population, while other ethnic groups include Hungarians, Bosniaks, Roma, and Croats. From a religious perspective, 84.6% of the population belongs to the Serbian Orthodox Church, while minority communities include Roman Catholics, Protestants, and Muslims. Regarding the educational structure, it is notable that around 17% of the population has higher education, while around 50% have completed secondary education. Serbia is experiencing negative migration trends, as many young people are leaving the country in search of better opportunities abroad. In 2019, about 66.5% of the population rated their health as good, 22.8% as average, and 10.7% as poor. Additionally, 71.3% of men rated their overall health as good, compared to 62.1% of women. There were notable differences in self-assessed health between urban residents, where 70.1% rated their health as good, and those in rural areas, with only 60.9% doing the same. Disparities were also evident between the wealthiest (77.7%) and the poorest (55.7%), as well as between the most educated (80.2%) and those with lower levels of education (45.1%) [91].

The study encompasses a broad range of regions across Serbia, including major urban centres like Belgrade and Novi Sad, as well as rural and isolated areas where access to emergency services might be more restricted. Moreover, this regional diversity facilitates a thorough evaluation of EMS across various environments, addressing the risks and evaluating the efficiency of the existing emergency preparedness and response strategies [7,12]. As well, considering Serbia’s recent experiences with natural hazards [92] and its ongoing efforts to enhance disaster readiness [90], the country serves as a significant case study for assessing the effectiveness of its emergency medical response systems.

According to the Law on Healthcare (“Official Gazette of the Republic of Serbia”, No. 25 of 3 April 2019, 92 of 27 October 2023), healthcare institutions can be established with funds from either public or private ownership. Furthermore, public healthcare institutions are established by the Republic of Serbia, autonomous provinces, or local government units, while private healthcare institutions can be established by legal or natural persons. On the other side, healthcare institutions can be established in the form of health centers, polyclinics, pharmacies, hospitals (general and specialized), health centers, institutes, public health institutes, clinics, institutes, clinical-hospital centers, and university clinical centers, as well as military healthcare institutions or sanitary units within the Serbian Armed Forces, following specific laws.

It is very important to mention that the healthcare network plan determines the number, structure, capacities, and spatial distribution of public healthcare institutions and their organizational units across levels of healthcare, as well as the organization of emergency medical services and other important issues related to the organization of the healthcare system in Serbia. The health centre is a primary-level healthcare institution that provides preventive care for all population groups, healthcare for children and women, general medicine, patronage services, home treatment, and palliative care. In public health centres, depending on the number of residents and their needs, emergency medical services are organized in accordance with the healthcare network plan. If emergency medical services are not provided by another public healthcare institution in the designated area, the health center is responsible for providing these services. At the secondary level of healthcare, inpatient and specialized services are provided by general and specialized hospitals (“Official Gazette of the Republic of Serbia”, No. 25 of 3 April 2019, 92 of 27 October 2023).

Based on the available data from 2020, according to [93] and the Regulation on the Healthcare Institutions Network Plan (“Official Gazette of RS”, Nos. 5/2020, 11/2020, 52/2020, 88/2020), in the AP Vojvodina region, there are currently 93 public healthcare institutions, including 10 pharmaceutical institutions operating 47 pharmacies, 284 private healthcare institutions, and 960 private pharmacies. In the Western Serbia region, there are 39 public healthcare institutions, organized into 26 legal entities, as well as 87 private healthcare institutions and 279 private pharmacies. The Šumadija and Central Serbia region has 55 public healthcare institutions, along with 196 private healthcare institutions and 564 private pharmacies. The Eastern Serbia region is the least developed in terms of private healthcare, with 31 public healthcare institutions, 90 private healthcare institutions, and 205 private pharmacies. Finally, the Southern Serbia region has 51 public healthcare institutions, 115 private healthcare institutions, and 346 private pharmacies (Table 1).

### 2.2. Sample Characteristics

The study included 172 participants drawn from various healthcare institutions actively involved in emergency medical services (EMS) across Serbia. A total of 198 participants from healthcare institutes were contacted to take part in the survey. Of these, 172 participants took the survey, and this led to a response rate of almost 86.9%. A significant portion, 81.4%, were in leadership roles within their institutions, while 13.37% comprised medical staff. Moreover, the remaining participants were administrative personnel (4.07%) and operational medical staff (1.16%). As for the type of institution, the majority (70.93%) were affiliated with public health centres, 22.67% worked in private healthcare facilities, and 6.40% were connected to hospitals. Participants’ experience levels showed diversity, with nearly half (49.42%) having worked in EMS for 5 to 10 years, 26.16% for less than 5 years, and 24.42% for more than 10 years. Gender distribution was fairly even, with 55.23% male and 44.77% female participants. Regarding education, 58.14% held a bachelor’s degree, 24.42% had earned a master’s degree, and 17.44% had completed high school (Table 2).

A notable 70.93% of participants had undergone one or more training sessions related to emergency medical services, while 29.07% had not received any such training. In their roles, 46.51% of participants identified as coordinators, 29.07% as first responders, and 24.42% as support staff. Additionally, 63.95% of the institutions reported having established mass casualty plans or procedures, while 36.05% did not have such measures in place. This diverse group underscores the varied levels of experience, roles, and readiness among healthcare institutions engaged in emergency medical services throughout Serbia (Table 2).

### 2.3. Questionnaire Design

In the first step, a communication was sent to the offices of all city mayors and municipal leaders, requesting that it be relayed to the appropriate healthcare institutions within their areas, specifically those responsible for providing Emergency Medical Services (EMS), such as EMS centres and health clinics. In response, 172 healthcare institutions (Public Health Centers and Hospitals) completed the survey.

The survey questionnaire (see Appendix A) has been carefully designed to collect in-depth insights into how emergency medical services (EMS) in Serbia are organized and operate. In addition to that, this extensive questionnaire is divided into seven primary sections, each targeting a different aspect of EMS, with a varying number of questions designed to extract precise information: (a) organizational structure and risk management of emergency medical services (27 questions); (b) resource allocation (staffing) and efficacy in emergency preparedness (10 questions); (c) communication systems and efficacy in coordinated response (17 questions); (d) reaction time for first-order emergencies (3 questions); (e) training (education) and preparedness for disaster response (6 questions); (f) funding for emergency medical services (EMS) (12 questions); (g) ambulance vehicles and equipment (5 questions); (h) emergency response and efficacy in urgent interventions, mass casualties (8 questions).

Each section is meticulously designed to provide a holistic overview of EMS capabilities and challenges, ensuring that all critical aspects of emergency preparedness and response are comprehensively addressed. Also, before the study commenced, a dedicated group of experts conducted an in-depth review of all the questions in the questionnaire. Moreover, this panel comprised professionals from various fields pertinent to emergency medical services and disaster preparedness, including specialists in healthcare management, public health policy, and disaster risk management. On the other side, their analysis aimed to guarantee that the questions were not only comprehensive and relevant but also reflective of the current socio-economic and political landscape. The experts also advised updating the questions to align with recent changes in healthcare policies and practices, acknowledging the dynamic nature of disaster risk management.

After the expert review, a pilot study was undertaken to test the revised questionnaire. Additionally, this preliminary study engaged a small group of participants from various healthcare institutions. Its goal was to evaluate the questionnaire’s functionality, uncover any issues with question interpretation, and assess the overall coherence and flow of the survey. The pilot study yielded valuable insights into the questionnaire’s practical application, highlighting areas where further refinement was needed. Feedback from participants was instrumental in enhancing the language and structure of the questions, ensuring they were clear and pertinent to the respondents’ experiences.

### 2.4. Analyses

The research utilized a range of statistical methods, such as Pearson’s correlation [94], multivariate linear regression [95], and chi-square tests [96], to analyze the data comprehensively. Initially, the analysis revealed a breach of the equal variance assumption, prompting the application of the Welch and Brown–Forsythe tests [97], which are well-suited for handling such deviations. To provide a clear overview of the dataset, descriptive statistical analysis was also conducted. The statistical tests were executed using a two-tailed approach with a significance level of *p* < 0.05, employing IBM SPSS Statistics (Version 26, New York, NY, USA). Additionally, the study assessed internal consistency across various subscales utilizing Likert scales, yielding promising outcomes. The study was conducted according to the guidelines of the Declaration of Helsinki [98] and approved by the Institutional Review Board of the Scientific–Professional Society for Disaster Risk Management and the International Institute for Disaster Research (protocol code 005/2024, 15 July 2024). Also, the authors acknowledge the use of Grammarly Premium (1.2.96) and ChatGPT 4.0 in the process of translating and improving the clarity and quality of the English language in this manuscript.

## 3. Results

The results of the study are presented in three dimensions: the predictors of risk and efficacy analysis of emergency medical response systems in Serbian healthcare; correlations and influences of demographic and socioeconomic factors on the perception of risk and efficacy analysis of emergency medical response systems in Serbian healthcare; and descriptive analysis parts: organizational structure and risk management of emergency medical services; resource allocation and efficacy in emergency (disaster) preparedness; communication systems and efficacy in coordinated response; emergency response times and efficacy in urgent interventions; training and preparedness for disaster response; and financial resources and administrative efficacy in emergency medical services.

### 3.1. The Predictors of Risk and Effectiveness Analysis of Emergency Medical Response Systems in Serbian Healthcare

The results from the multivariate regression analysis regarding the organization of EMS reveal that both the organization of working hours (*β* = 0.035) and shift work (*β* = 0.042) are the most significant predictors, together explaining 1.9% of the variance. Other factors, such as EMS teams working only in the clinic and financial resources, did not show statistically significant effects on EMS organization. This model (*R*^2^ = 0.019, *Adj. R*^2^ = 0.006, *F* = 2.78, *t* = 59.87, *p* < 0.05) explains 1.9% of the variance in the organization of EMS, considering all the independent variables included in the analysis (Table 3).

For the number of EMS points performed, the analysis indicates that the organization of shift work (*β* = −0.045) and working hours (*β* = −0.037) are significant predictors, accounting for 2.0% of the variance. In contrast, other variables like ambulance vehicles and financial resources did not contribute significantly to the model. This regression model (*R*^2^ = 0.020, *Adj. R*^2^ = 0.008, *F* = 3.15, *t* = 62.14, *p* < 0.05) explains 2.0% of the variance in the number of EMS points performed (Table 3).

When it comes to service area coverage, the regression results suggest that none of the predictors were statistically significant. Although the model (*R*^2^ = 0.027, *Adj. R*^2^ = 0.015, *F* = 3.50, *t* = 63.21, *p* ≥ 0.05) explains 2.7% of the variance in service area coverage, this relationship was not found to be statistically significant (Table 3).

In the case of the EMS doctors, the presence of EMS teams working only in the clinic (*β* = 0.07) emerged as a significant predictor, explaining 3.5% of the variance. However, other factors, such as shift work and financial resources, did not show statistical significance. This model (*R*^2^ = 0.035, *Adj. R*^2^ = 0.022, *F* = 3.88, *t* = 64.45, *p* < 0.05) accounts for 3.5% of the variance in the total number of EMS doctors (Table 3).

Lastly, the regression analysis related to plans/procedures for mass casualties indicates that ambulance vehicle availability (*β* = 0.075) and financial resources (*β* = 0.033) are significant predictors, explaining 4.1% of the variance. Other variables, such as the organization of working hours, were not significant in this context. This model (*R*^2^ = 0.041, *Adj. R*^2^ = 0.030, *F* = 4.25, *t* = 65.72, *p* < 0.05) explains 4.1% of the variance in the presence of plans or procedures for mass casualties (Table 3).

These findings offer valuable insights into the essential factors driving the success of EMS organizations, particularly emphasizing the critical role of managing working hours and shift schedules, which have a direct impact on the efficiency of emergency medical interventions during disaster situations.

### 3.2. Correlations and Influences of Demographic and Socioeconomic Factors on the Perception of Risk and Effectiveness Analysis of Emergency Medical Response Systems in Serbian Healthcare

Based on Pearson’s correlation results, there is a statistically significant correlation between the EMS organization and several key variables. These include the total number of EMS doctors (*p* = 0.000), the number of emergency medicine specialists (*p* = 0.000), the number of doctors in emergency medicine training (*p* = 0.000), the number of general practitioners in EMS (*p* = 0.001), the number of permanent EMS ambulance drivers (*p* = 0.000), the number of day shift teams on weekdays (*p* = 0.000), the number of night shift teams on weekdays (*p* = 0.000), the maximum distance from EMS headquarters to the hospital (*p* = 0.002), the gender distribution of male doctors (*p* = 0.000), the gender distribution of female doctors (*p* = 0.009), the number of male emergency medicine specialists (*p* = 0.000), and the number of female emergency medicine specialists (*p* = 0.000). On the other hand, Pearson’s correlation analysis revealed no statistically significant correlation between EMS organization and other variables (Table 4).

Additionally, a statistically significant correlation was identified between the number of EMS points performed and the variable “doctors with verified limited working capacity” (*p* = 0.033). However, no significant correlations were found with other variables (Table 4). Further analyses revealed that as the total number of EMS doctors grows, the organization of EMS services tends to become more structured and effective. A similar pattern is observed with the increase in emergency medicine specialists, where their presence boosts both specialization and the system’s ability to respond swiftly to emergencies. As more doctors enter emergency medicine training, the EMS organization gains strength, which signals a clear emphasis on preparing for the future. Additionally, having more general practitioners involved in EMS correlates with improved coverage and a more solid organizational structure, while a greater number of permanent EMS ambulance drivers leads to noticeable gains in operational efficiency and the overall organization of EMS.

Moreover, adding more day shift teams during weekdays results in better resource management and a more organized EMS system. Similarly, an increase in night shift teams enhances the system’s flexibility, allowing it to meet nighttime demands more effectively. Also, the analysis also pointed out that as the distance between EMS headquarters and the hospital increases, the organization becomes more structured to ensure a timely response and smooth patient transfer. Regarding gender distribution, a higher percentage of male doctors is linked to a more structured EMS organization, which might be influenced by staffing patterns. On the other hand, an increase in female doctors brings balance to the organization, possibly reflecting a more diverse range of roles within the staff. When the number of male emergency medicine specialists rises, the organization becomes more specialized, focusing on emergency care, and a similar effect is seen with female specialists, where their growing presence contributes to improved preparedness and organization within EMS. In addition, the data indicate that as the number of doctors with verified limited working capacity increases, there is also an increase in the number of EMS points performed, suggesting that staffing adjustments have been made to accommodate these limitations.

Recognizing the significant correlations between demographic and socioeconomic factors and EMS organization paves the way for further exploration into optimizing human resources and resource allocation. This research could ultimately enhance disaster response efforts.

The results of the Chi-square test highlight a statistically significant correlation between the organization of emergency medical services (EMS) and several critical variables. Notably, there is a strong relationship between EMS organization and how EMS activities are conducted (*p* = 0.001), the number of points where these activities take place (*p* = 0.005), and the structure of working hours (*p* = 0.001). The organization of shift work (*p* = 0.001) and the presence of a dedicated EMS team working exclusively in the clinic (*p* = 0.004) also show significant correlations (Table 5).

In addition, the number of ambulance transport teams per shift during the day (*p* = 0.001), night (*p* = 0.001), and weekends (*p* = 0.001) is significantly tied to EMS organization. Other relevant factors include the composition of the ambulance transport team (*p* = 0.001), on-call duties in cases where the team needs to leave the territory (*p* = 0.003), and whether the regular shift workload includes additional responsibilities (*p* = 0.001). Further significant correlations emerged regarding the number of doctors in EMS (*p* = 0.006), the regularity of annual medical examinations for doctors (*p* = 0.002), limited work capacity (*p* = 0.005), and the number of ambulance drivers (*p* = 0.001). Communication-related aspects such as having a separate phone number for ambulance transport (*p* = 0.007), call identification features (*p* = 0.018), the presence of a call recorder (*p* = 0.003), and recording calls on this system (*p* = 0.001) also demonstrated significant relationships (Table 5).

Moreover, the condition of radio repeaters (*p* = 0.008), the installation of radio stations in ambulances (*p* = 0.001), and having a power supply backup for the radio system in case of outages (*p* = 0.001) were all significantly correlated with EMS organization. Lastly, factors such as maintaining a dedicated communication channel with the police (*p* = 0.005) and firefighters–rescuers (*p* = 0.003), monitoring response times during interventions (*p* = 0.001), and training for emergency medicine doctors (*p* = 0.004) and nurses (*p* = 0.003) also showed significant associations (Table 5).

Further analysis shows that institutions with well-organized EMS systems exhibit a higher level of efficiency in conducting emergency medical activities, particularly when operating across multiple locations (27.3%) compared to single-point operations (20.3%). This ability to manage multiple service points ensures that resources are distributed evenly, reducing response times and enhancing the quality of care provided. The flexibility that comes from organizing EMS across various points (41.1%) further strengthens these institutions, enabling them to allocate staff and resources more effectively to meet community needs (Table 5).

The organization of working hours, including structured shifts, plays a crucial role in maintaining continuous service and adapting to varying demands. Institutions that implement structured 8 h shifts (24.4%) or other types of shifts (55.8%) can ensure that trained personnel are always available to respond to emergencies. A focus on shift work (42.4%) also helps institutions keep EMS teams well-rested and ready for emergencies at all times, reducing burnout and improving the overall quality of service provided. Specialized EMS teams working exclusively in clinics (28.9%) enhance clinic-based interventions, with staff trained to handle specific medical scenarios more effectively. Similarly, institutions that allocate more teams for ambulance transport during day shifts (43.5%) or night shifts (43.0%) can manage high-demand periods with greater efficiency, ensuring prompt responses to emergency calls. This flexibility extends to weekends and holidays, where well-organized EMS teams (47.1%) maintain uninterrupted service even during peak times (Table 5).

Effective resource management is vital for institutions with dedicated ambulance transport teams (34.7%) and well-organized on-call duty systems (42.4%), particularly when teams must leave their designated areas. By balancing regular shift workloads (48.6%), these institutions ensure that EMS teams are not overwhelmed and can continue delivering consistent care. Moreover, institutions with a higher number of doctors in their EMS teams (42.4%) and those that conduct regular medical examinations for staff (26.2%) are better equipped to maintain a healthy, capable workforce (Table 5).

Also, reliable communication systems are essential for EMS operations. Institutions with separate phone lines for ambulance transport (23.3%) and call identification capabilities (45.3%) are better organized, enabling them to handle emergency calls efficiently. Established protocols for receiving calls (53.2%) and call recording capabilities (42.0%) further enhance the quality of service by ensuring that communications are documented and reviewed. Additionally, equipping ambulances with radio stations (58.4%) and maintaining functional radio repeaters (26.2%) guarantees that communication channels remain operational during emergencies (Table 5).

Institutions that foster collaboration with other emergency services demonstrate improved coordination during crises. Those with dedicated communication channels with police (26.2%) and firefighters (26.2%) can work more effectively with these agencies during joint operations, ensuring timely medical support in various emergency scenarios. Regular monitoring of intervention reaction times (39.5%) and specialized training for emergency medicine doctors and nurses (42.4%) also contribute to the institution’s ability to handle a wide range of medical emergencies (Table 5).

Finally, institutions that receive additional financial resources for healthcare (40.7%) are better positioned to expand services, hire more staff, and maintain well-equipped ambulance vehicles (42.4%). This financial support, coupled with vehicles for mass casualty incidents (34.7%) and written plans for handling such events (48.8%), ensures that EMS teams are prepared for large-scale emergencies. Regular exercises and drills (41.8%) with other first responders also help these institutions refine their response strategies, ensuring that all personnel are ready to act efficiently during real-world disasters (Table 5).

The results from the Chi-square test reveal statistically significant correlations between EMS employee training and a range of key variables. Notably, there is a strong correlation between EMS employee training and the conducting of EMS activities (*p* = 0.001), as well as with the organization of working hours (*p* = 0.002) and shift work (*p* = 0.001). Additionally, significant correlations were identified between EMS employee training and the number of ambulance transport teams working both day (*p* = 0.001) and night (*p* = 0.003) shifts, along with those working on weekends (*p* = 0.001). The data also shows significant relationships between EMS employee training and the organization of on-call duty when teams are outside their designated areas (*p* = 0.001), the implementation of protocols and procedures for receiving calls (*p* = 0.000), and the monitoring of response times during interventions (*p* = 0.000). Moreover, there are strong correlations with the availability of dedicated communication channels with the police (*p* = 0.000) and firefighters (*p* = 0.002), as well as with the financial resources allocated to healthcare (*p* = 0.003). EMS employee training also shows significant correlations with the presence of triage tags (*p* = 0.002), exercises for responding to mass casualty incidents (*p* = 0.001), and joint exercises with other first responders (*p* = 0.005). For the remaining variables, no statistically significant correlations were found (Table 5).

Further analysis shows that conducting EMS activities is notably more effective in institutions where employees have undergone formal training at recognized centers. These institutions exhibit a higher level of operational readiness, ensuring their staff is well-prepared to handle various medical emergencies. This preparation is evident in their ability to allocate tasks more efficiently, reducing the risk of errors during critical incidents. Also, when it comes to organizing working hours, institutions with formally trained staff are more likely to implement structured shifts (41.9%). This structured approach allows for better shift management, ensuring that trained personnel are available around the clock to handle emergencies. This leads to more consistent and dependable service delivery.

Similarly, institutions with trained EMS personnel are more likely to utilize shift work (42.4%), which supports continuous service provision. This systematic organization of shifts allows them to respond effectively to increased demand during peak periods, ensuring that qualified professionals are always present. For teams working exclusively in clinics, training in established centres correlates with better organizational efficiency (28.9%). Such institutions are more adept at handling clinic-based interventions, as their specialized training equips staff with the necessary skills to manage specific medical scenarios. Resource allocation during day shifts, particularly for ambulance transport, is more efficient in institutions with trained EMS staff (43.5%). This allows for more effective use of teams during busy hours, facilitating quicker responses to disasters.

At night, these institutions also excel in organizing shifts for ambulance transport (43.0%), ensuring adequate staffing during off-peak hours. This ability to maintain round-the-clock coverage helps meet emergency transport needs effectively. During weekends and holidays, institutions with trained staff are better equipped to manage ambulance transport services (47.1%), ensuring they can handle high demand without sacrificing care quality. When it comes to on-call duties, particularly when teams need to leave their designated areas, institutions with trained EMS staff demonstrate better management (42.4%). These organizations can maintain sufficient coverage, even when on-call teams are deployed to other locations.

Regular shift workloads are more effectively managed in institutions with trained personnel (48.6%). This allows them to balance emergency response with routine medical tasks without overwhelming their teams. In terms of staffing, institutions with more trained EMS personnel tend to have a more balanced distribution of doctors across teams (42.4%), ensuring that medical expertise is available whenever needed, thereby enhancing the quality of care provided. Institutions with dedicated ambulance transport phone lines (55.0%) are also more common where staff have received formal training. This specialization enhances their ability to coordinate emergency responses and streamline communication.

The implementation of call identification systems is more prevalent in institutions with trained EMS staff (45.3%), improving their ability to direct resources efficiently to where they are most needed, which shortens response times. Furthermore, institutions with trained personnel are more likely to have established protocols for handling emergency calls (53.2%). These protocols ensure standardized call handling, minimizing the risk of miscommunication or delays in critical situations. The presence of call recording devices is another feature more commonly seen in institutions with trained EMS staff (42.0%). These devices provide valuable documentation and allow for quality review, which can be essential for both performance assessment and legal purposes (Table 5).

Reliable communication with teams in the field is another advantage seen in institutions with trained personnel, where the use of radio systems is more frequent (46.6%). This ensures real-time coordination and response adaptability in the field. Ambulances in institutions with trained staff are more likely to be equipped with radio stations (58.4%), which facilitates continuous communication and improves the speed of coordination during emergency responses. Moreover, institutions with trained employees are more likely to have backup power systems for their radio equipment (42.4%), ensuring uninterrupted communication even during power outages, which is vital for maintaining service continuity in emergencies. Dedicated communication channels with the police are another feature more commonly found in institutions with trained staff (56.7%). This enhances coordination during joint operations, ensuring effective collaboration between EMS teams and law enforcement during disasters.

Monitoring reaction times to interventions is also more common in institutions with trained EMS employees (47.9%). This practice enables these organizations to evaluate and improve their response times, enhancing overall service delivery. Dedicated communication lines with firefighters are prevalent in institutions with trained EMS personnel (56.7%), facilitating better coordination during fire-related emergencies and ensuring that medical support is promptly provided. Institutions that prioritize training for their emergency medicine doctors tend to have better-prepared staff overall (42.4%), with the latest medical skills and knowledge needed to address a wide range of emergency situations effectively. The same is true for institutions that invest in the training of emergency medicine nurses (42.4%). This ensures nursing staff is well-equipped to handle high-pressure situations and deliver high-quality care during emergencies.

Additional financial resources allocated to healthcare are more common in institutions with trained EMS personnel (40.7%). This funding supports investment in training programs, leading to an expanded workforce and improved service delivery. Ambulance fleets in institutions with trained EMS staff are more likely to be well-maintained (42.4%), ensuring their readiness for immediate deployment in emergency scenarios and enhancing the overall responsiveness of the institution. Specialized vehicles for mass casualty events are also more common in institutions with trained EMS personnel (34.7%). This training enables staff to effectively manage large-scale emergencies, particularly in terms of the logistics of transporting multiple patients.

Institutions with trained staff are more likely to have written plans and procedures in place for mass casualty incidents (48.8%). These protocols ensure that all personnel are well-prepared for large-scale emergencies, enhancing their readiness and response capabilities. Triage tags, used to prioritize patients in mass casualty situations, are more readily available in institutions with trained EMS personnel (34.7%). This ensures that patients in the most critical condition receive attention first, improving the overall efficiency of care during such events. Regular drills and exercises for responding to mass casualty situations are more common in institutions with trained staff (41.8%). These exercises help teams refine their procedures and ensure they are ready to act quickly and effectively in real emergencies. Joint exercises with other first responders, such as police and firefighters, are also more frequently conducted in institutions with trained EMS personnel (41.8%). These collaborative exercises improve the coordination between different emergency services, enhancing the overall effectiveness of joint responses during major incidents.

Similarly, the Chi-square test results demonstrate statistically significant correlations between plans and procedures for mass casualty events and several critical variables. These include conducting EMS activities (*p* = 0.001), the organization of working hours (*p* = 0.000), and the presence of ambulance transport teams working during both day (*p* = 0.000) and night (*p* = 0.000) shifts. Additionally, significant correlations were observed between mass casualty event planning and the organization of on-call duty (*p* = 0.000), as well as the existence of protocols and procedures for receiving calls (*p* = 0.000). There is also a strong correlation between the presence of radio communication equipment in ambulances (*p* = 0.000) and the monitoring of response times during interventions (*p* = 0.005). Finally, plans for mass casualty events are significantly associated with the availability of vehicles for such incidents (*p* = 0.000), the presence of triage tags (*p* = 0.045), and the organization of joint exercises with other first responders (*p* = 0.000). No statistically significant correlations were identified for the remaining variables (Table 5).

Furthermore, results show that institutions that have written plans and procedures for mass casualty events demonstrate greater success in conducting EMS activities. These institutions are more likely to provide services from multiple dislocated points (55.9% for 47 institutions with plans) compared to those without such plans (55.3% for 21 institutions). The structured approach afforded by these plans helps streamline operations across various locations, ensuring a broader reach and better preparedness for emergencies. The organization of working hours also benefits from the existence of mass casualty plans. Institutions with these plans are more inclined to implement shift work (52.4% for 44 institutions) compared to those without plans (60.5% for 23 institutions). This structure facilitates continuous service provision, allowing institutions to maintain operational efficiency and ensure staff availability at all times, particularly during high-demand periods.

When it comes to ambulance transport teams, institutions with mass casualty plans excel in organizing both day and night shifts. These institutions effectively allocate resources during peak times (58.3% for day shifts and 51.2% for night shifts), compared to those without plans. Additionally, the existence of such plans enables better management of on-call duties (60.7% for 51 institutions with plans), especially when teams need to operate outside their designated territories, ensuring uninterrupted coverage.

Institutions with mass casualty plans are also more likely to have established protocols and procedures for receiving emergency calls (78.6% for 66 institutions). This formalized process enables them to manage critical situations more effectively than institutions without plans (39.5% for 15 institutions). Furthermore, these institutions are better equipped with radio communication systems in ambulances (79.8% for 67 institutions), which enhances coordination during interventions and ensures seamless communication between teams.

Monitoring response times is another area where institutions with mass casualty plans outperform. By tracking response times (63.1% for 53 institutions), they can evaluate their efficiency and make necessary adjustments to improve overall performance. Moreover, these institutions are more likely to have specialized vehicles for mass casualty incidents (10.7% for 9 institutions), making them better prepared to handle large-scale emergencies compared to institutions without plans (2.6% for 1 institution). Lastly, the presence of mass casualty plans correlates with the availability of triage tags (40.5% for 34 institutions) and the organization of joint exercises with other first responder services (58.3% for 49 institutions). These factors contribute to enhanced coordination and preparedness during emergencies, ensuring that all agencies involved are well-equipped to respond effectively to mass casualty events.

### 3.3. Organizational Structure and Risk Management of Emergency Medical Services (EMS)

The study uncovers a variety of approaches to structuring Emergency Medical Services (EMSs), each influencing the efficiency and effectiveness of emergency response capabilities in distinct ways. To be specific, the organizational structure of EMS within healthcare facilities shows notable diversity. A substantial portion, 46.04%, is integrated within dedicated EMS departments in health centres, a model that supports focused management of emergency care. Another 33.81% operate within general medical services, where regular medical staff, including doctors and health workers, manage emergencies as part of their routine duties. In contrast, 15.11% of facilities have established EMS as separate organizational units within the broader medical framework, emphasizing the allocation of specific resources for emergency services. A smaller fraction, 3.60%, functions as specialized entities like Institutes for Emergency Medical Services, while only 1.44% lack an organized EMS system, indicating areas that may require service expansion (Table 6).

Most EMS activities occur within healthcare facilities (45.3%), utilizing existing infrastructure to facilitate emergency operations. Meanwhile, 26.7% of EMS activities are centralized in a single location, and 7.6% are distributed across multiple sites, aiming to enhance coverage and accessibility. The data also show that EMS activities are predominantly concentrated in one or two locations, accounting for 88.89% of operations, reflecting a centralized management approach. A smaller percentage of facilities operate across multiple locations, with 4.44% having 3 to 10 points and 1.11% reporting more than 11 points, showcasing varying degrees of decentralization to meet regional needs (Table 6).

Regarding working hours, 55.2% of facilities employ shift work to ensure continuous service delivery, while 44.8% use rotating shifts to balance workloads among staff. Among these, 80.2% of services operate on 12 h shifts, the most common scheduling model, whereas 11.6% and 8.1% adhere to 8 h shifts or alternative configurations, respectively, illustrating adaptability to operational demands and workforce preferences. In terms of specific shift patterns, 51.85% of facilities follow a schedule with a day shift, a subsequent 24 h rest, and a night shift with 72 h off, balancing work demands with adequate rest. Other patterns include a day shift followed by 48 h off (24.44%) and a 48 h rest period after each shift (23.70%) (Table 6).

During daytime shifts on weekdays, a single-team configuration is prevalent in 50.6% of cases, facilitating streamlined operations. Conversely, 16.9% of facilities employ two teams, and 3.5% use three or more teams to address higher demand or specific challenges. Additionally, 27.9% of services utilize special configurations tailored to unique needs. For nighttime shifts on weekdays, 48.3% of facilities operate with one team, a common practice for maintaining service readiness. Meanwhile, 16.3% use two teams, 11.0% deploy three or more teams, and 23.2% implement special configurations to meet nighttime requirements. Only 1.2% report having no teams during night shifts, possibly due to low demand or reliance on on-call staff. Regarding healthcare management plans, 28.5% of facilities have teams dedicated solely to clinic operations, focusing on non-emergency services. In contrast, 49.4% do not differentiate between clinic and EMS teams, suggesting an integrated approach, while 22.1% report varied organizational structures that preclude direct comparison (Table 6).

Clinic team configurations for daytime shifts predominantly involve one team (64.0%), optimizing resource allocation for clinic operations. A smaller segment employs two teams (8.1%) or three or more teams (7.0%), reflecting complex operational demands. At nighttime, 72.1% of clinics maintain operations with one team, whereas 15.1% report no teams, possibly relying on emergency services for critical care during these hours. Finally, transport by a team composed of a medical nurse-technician and driver accounts for 19.2% of facilities, emphasizing a lean team setup designed for specific transport needs while balancing resource efficiency with the ability to address urgent situations (Table 6).

This examination of EMS structures highlights the importance of standardizing organizational models and refining protocols, which could strengthen emergency response capabilities across various regions in Serbia.

This next analysis delves into the operational capabilities and strategic readiness of emergency medical services (EMSs), examining medical transport teams, geographical coverage, and how seasonal population changes affect service delivery. Medical transport teams are typically organized with a single team, as reported by 43.0% of responses, allowing for streamlined patient transport operations. A smaller segment, 14.0%, deploys two teams, while configurations involving three (4.1%) and four teams (4.1%) indicate facilities with higher demand or specific operational requirements. A few facilities use five teams (1.2%) or more (1.7%), reflecting substantial transport capabilities. On the other side, the reliance on single-team configurations increases to 47.7% for overnight transport needs. In 19.8% of cases, no teams are reported, suggesting minimal demand or alternative staffing strategies, such as on-call services. Only 5.8% utilize two teams, indicating a targeted approach to nighttime operations (Table 7).

The composition of medical transport teams often includes a vehicle driver (33.1%) or a nurse-technician paired with a driver (29.7%), highlighting the lean operational structures in place. However, 37.8% of teams are more comprehensive, consisting of a doctor, nurse/technician, and driver, ensuring thorough patient care during transport. Other configurations, such as driver-only or driver with occasional medical staff (16.9%), and teams assembled based on specific needs (14.0%), reflect flexibility in adapting to different patient conditions. Teams vary depending on patient needs (20.9%), showcasing the adaptability necessary for appropriate care (Table 7).

Across facilities, 43.0% report established emergency readiness, while 57.0% lack specific plans, indicating a need for improved strategic planning. Similarly, 48.3% of facilities have organized preparedness for vehicle drivers, whereas 51.7% do not, highlighting an opportunity to enhance emergency transport efficiency. The average holding time for medical teams in higher-level centers varies, with most teams spending 61–120 min (18.0%) or 31–60 min (15.7%) at these locations, reflecting the time needed for patient handovers and administrative tasks. Transport teams usually spend 31–60 min (18.6%) at centers, indicating efficient turnover for prompt service resumption (Table 7).

Healthcare service coverage (HMP) ranges widely, with the most common area being 300–400 km^2^ (20.3%). Many facilities cover areas between 100–200 km^2^ (10.5%) and 400–500 km^2^ (10.5%), demonstrating varying regional service demands. The typical territory diameter is 30–60 km (60.5%), indicating broad reach within the healthcare system. Also, the maximum distance from HMP headquarters to hospitals is primarily 25–50 km (31%), suggesting strategic facility placement for timely patient transport. For tertiary centers, the most common distances are 60–90 km (23.8%), reflecting the distribution of specialized services. Institutions covering parts of a highway report mixed responses: 50.0% indicate no coverage, 26.2% confirm coverage, and 23.8% find it non-applicable. Highway access is crucial for efficient logistics and rapid emergency site access (Table 7).

Population changes in HMP jurisdictions are noted by 48.8% of respondents, emphasizing the impact of seasonal influxes on healthcare demand. Increases typically involve fewer than 1000 people (40.7%) or 1000–5000 people (37.2%), often due to tourism and migration (37.8%) or temporary residents (19.8%). Beyond urgent care, 65.1% of facilities report regular shift workloads that encompass various healthcare services, managed primarily by regular staff (75.0%) rather than on-call duty (25.0%). During night shifts for urgent care, transport teams mainly consist of a full medical team (73.8%), emphasizing comprehensive care during critical transport operations. This configuration reflects a commitment to delivering high-quality patient care in emergencies at all times (Table 7).

### 3.4. Resource Allocation and Effectiveness in Emergency (Disaster) Preparedness

In emergency medical service (EMS) facilities, a significant number, precisely 25.58%, function with a moderate staffing model of 3 to 5 doctors. This configuration appears to be common for handling emergency care effectively. Following this, 18.02% of facilities have teams of 6 to 8 doctors, with another 18.02% maintaining 9 to 11 doctors. These figures suggest a preference for medium-sized teams capable of efficiently managing disasters. Notably, 11.63% of EMS facilities operate with a smaller team of just 0 to 2 doctors, which could indicate challenges in staffing for some institutions. On the other hand, 11.05% boast a slightly larger team of 12 to 15 doctors, and a smaller fraction, 8.72%, have over 15 doctors, likely reflecting those with a higher capacity for complex cases (Table 8).

When it comes to specialists, a large portion, 51.74%, of EMS facilities have only 0 to 2 specialists, highlighting the difficulty in hiring specialized personnel. A moderate number, 15.70%, report having 3 to 5 specialists, while fewer institutions fall into the categories of 6 to 8, 9 to 11, 12 to 15, and over 15 specialists, at 4.07%, 1.74%, 1.16%, and 2.33%, respectively. This distribution indicates that specialists, while present, are generally concentrated in a limited number of facilities (Table 8).

Among doctors undergoing emergency medicine training, most EMS institutions (67.44%) have 0 to 2 doctors in training, pointing to opportunities for growth in workforce development. A smaller segment, 4.65%, reports 3 to 5 doctors in training, with even fewer institutions having 6 to 8 doctors at 1.16% and 9 to 11 doctors at 0.58%. Looking at EMS doctors who are specialists in general medicine, 58.14% of institutions are staffed with 0 to 2 specialists, indicating a tendency toward employing generalists. Another 13.37% have 3 to 5 specialists, while a minor segment, 2.33%, employs 6 to 10 specialists. Only 0.58% have more than 10 specialists, underscoring the scarcity of high specialization within EMS (Table 8).

The distribution of general medicine doctors in EMS is such that 43.02% of institutions operate with 0 to 4 doctors, followed by 24.42% with 5 to 9 doctors. This suggests a balanced staffing approach, with fewer facilities (6.40%) maintaining 10 to 19 doctors and none exceeding 20, reflecting a structured limitation on general practitioners. In terms of additional medical specialties, 54.7% of institutions incorporate these to broaden the range of services offered. Conversely, 25.0% report that such integration is not applicable, possibly due to strategic decisions or institutional focus. An additional 20.3% lack other specialties entirely, which might indicate limitations in diversifying services (Table 8).

Among medical specialties, 49.4% of institutions are dedicated to specialized fields, including gynecology and pediatrics, showcasing the breadth of available expertise. General medicine makes up 17.4% of specialties, playing a crucial role in foundational healthcare. Diagnostics and laboratory services represent 23.3%, emphasizing their importance in medical facilities. Surgical specialties are found in 5.8% of institutions, with other less common specialties making up 4.1%, highlighting the varied medical landscape in these settings (Table 8). These findings underscore the diverse distribution of medical staff and specialties within EMS, with a strong focus on moderate-sized teams and generalists, while also identifying potential areas for specialist expansion and service diversification.

The findings suggest that the strategic readiness of EMS, particularly in resource allocation and team configuration, is vital for maintaining operational efficiency during high-demand periods and large-scale emergencies. Strengthening these areas could significantly improve EMS responsiveness and overall disaster preparedness.

This analysis sheds light on staffing patterns within emergency medical services (EMS), revealing strengths and potential gaps in gender representation and specialization. In terms of male doctors, most institutions (41.3%) employ between 0 and 5 doctors, indicating a prevalent staffing level. Meanwhile, 15.7% of institutions have 6 to 10 male doctors, and 12.2% maintain 11 to 20, reflecting moderate staffing levels across many facilities. A smaller segment of institutions, 2.3%, employs 21 to 30 male doctors, with only 2.9% exceeding 30, suggesting that larger teams of male doctors are relatively uncommon (Table 9).

Regarding female doctors, 30.2% of institutions have 0 to 5 doctors, marking the most common staffing range for women in the field. Meanwhile, 19.2% employ 6 to 10 female doctors, and 17.4% have 11 to 20, showing a somewhat more balanced distribution compared to male doctors. Only 4.1% of institutions have 21 to 30 female doctors, while 3.5% have more than 30, highlighting a slightly more constrained presence of female doctors in larger numbers (Table 9).

For male specialists in emergency medicine, 62.2% of institutions employ 0 to 2 specialists, emphasizing a significant reliance on a minimal number of male specialists. Only 9.3% have 3 to 5 male specialists, with even smaller proportions, 2.9%, 0.6%, and 0.6%, in the categories of 6 to 10, 11 to 15, and over 15 male specialists, respectively. Female specialists in emergency medicine predominantly fall within the 0-to-2 category as well, with 65.7% of institutions reporting this number. About 5.8% of institutions employ 3 to 5 female specialists, and even smaller percentages, 1.2%, 0.6%, and 0.6%, report having 6 to 10, 11 to 20, and more than 20 female specialists, respectively, suggesting limited presence at higher levels (Table 9).

In terms of male doctors specializing in emergency medicine, 56.4% of institutions report having no specialists, indicating a lack of specialization in many facilities. A smaller group, 14.0%, has 1 to 2 male specialists, and only 3.5% have 3 to 5, pointing to potential areas for growth. Conversely, female doctors specializing in emergency medicine show similar trends, with 54.1% of institutions lacking specialists. Also, about 16.3% have 1 to 2 female specialists, while only 2.9% report having 3 or more, suggesting that specialization among female doctors is similarly limited (Table 9).

For male general medicine specialists, 44.8% of institutions report no specialists, and 24.4% have 1 to 2 specialists, indicating a trend toward low specialization in this area. Only 4.7% of institutions have 3 or more male specialists, suggesting room for increased specialization. Female general medicine specialists are slightly more prevalent, with 35.5% of institutions having no specialists and 23.8% employing 1 to 2. Approximately 9.9% of institutions employ 3 to 5 female specialists, while 4.1% have 6 or more, indicating a more significant presence compared to their male counterparts (Table 9).

For male general medicine doctors, 48.8% of institutions have 0 to 2 doctors, highlighting a primary staffing level. Meanwhile, 19.2% of institutions employ 3 to 5 male doctors, and only 5.2% have 6 to 10, with a minimal 0.6% exceeding 10, reflecting limited higher staffing levels. On the other side, for female general medicine doctors, 29.1% of institutions employ 0 to 2 doctors, showing a slightly lower presence than male doctors in this category. About 20.3% have 3 to 5 doctors, and 14.0% employ 6 to 10, with 9.9% reporting 11 or more female doctors, indicating a broader distribution among female general practitioners (Table 9).

Among male nursing staff with higher education, 60.5% of institutions employ 0 to 1 staff, indicating limited numbers of highly educated male nurses. Only 9.9% have 2 to 4 male nurses, and 2.9% employ 5 or more, suggesting potential areas for expansion. Female nursing staff with higher education show a similar trend, with 55.2% of institutions having 0 to 2 staff. About 9.3% employ 3 to 5, and another 9.3% have 6 or more, reflecting a slightly higher presence compared to their male counterparts (Table 9).

For male nursing technicians with secondary education, 44.2% of institutions employ 0 to 5 technicians, indicating a common staffing range. Meanwhile, 12.8% have 6 to 10, with smaller percentages of 7.6% and 9.3% employing 11 to 20 and 21 or more technicians, respectively. Female nursing technicians with secondary education are less prevalent, with 20.3% of institutions having 0 to 5 technicians. A higher proportion, 23.8%, have 6 to 10, while 20.3% employ 11 to 20, and smaller percentages employ more, indicating a more even distribution of female nursing technicians across different staffing levels (Table 9). Overall, these data suggest that while gender distribution in EMS is generally balanced, opportunities exist to enhance specialization, particularly among male and female specialists in emergency and general medicine. Additionally, increasing the presence of both male and female nursing staff could further support comprehensive healthcare delivery.

For doctors under the age of 30, a substantial majority of institutions, about 61.0%, report having just 0 to 1 doctor. This suggests a relatively low presence of younger doctors within the workforce. A smaller portion, 11.0%, employs between 2 and 5 doctors, while only 1.2% have 6 or more, indicating challenges in recruiting or retaining young doctors. In contrast, among doctors aged 30 to 55, there is a more balanced distribution. Here, 22.7% of institutions report having 0 to 5 doctors and another 22.7% have 6 to 10. The largest group, 27.9%, has 11 to 20 doctors, reflecting that mid-career professionals are the most prevalent in this age group. Smaller proportions, 8.1% and 4.1%, have 21 to 30 and 31 or more doctors, respectively, indicating a decline in higher numbers. For doctors over the age of 55, 49.4% of institutions have 0 to 5 doctors, suggesting a transition toward retirement. Approximately 11.0% of institutions report having 6 to 10 doctors, while 13.4% have 11 or more, underscoring the continued presence of experienced doctors nearing the end of their careers (Table 10).

Among nursing technicians under 30 with secondary education, 51.7% of institutions employ 0 to 1 technician, highlighting limited entry-level opportunities. Meanwhile, 15.7% employ 2 to 4 technicians, and only 5.8% have 5 or more, indicating a focus on retaining more experienced staff. Nursing technicians aged 30 to 55 show a diverse distribution. Around 16.9% of institutions have 0 to 5 technicians, while 19.2% have 6 to 10. Notably, 14.0% employ 11 to 15 technicians, and 17.4% have 21 or more, reflecting a strong presence of mid-career professionals. For those over the age of 55, 34.9% of institutions employ 0 to 2 technicians, suggesting a trend toward retirement. Meanwhile, 21.5% have 3 to 5 technicians, with smaller percentages, 10.5% and 6.4%, employing 6 to 10 and 11 or more, respectively, highlighting the retention of experienced staff (Table 10).

Among doctors, 61.6% report no verified limitations in work capacity, indicating a generally healthy workforce. However, 10.5% have minor limitations, and 1.7% have significant ones, suggesting areas for intervention to maintain productivity. Compliance with annual medical examinations is relatively high among doctors, with 47.1% meeting the requirements. However, 26.7% do not comply, revealing potential gaps in regulatory adherence. For medical nurses and technicians, 45.3% comply with annual examinations, while 28.5% do not, indicating similar compliance challenges as seen with doctors (Table 10).

Among ambulance drivers, compliance with annual medical examination requirements is strong, with 64.5% adhering to standards. Nonetheless, 9.3% do not comply, highlighting areas for improvement. Regarding work capability, 69.2% of ambulance drivers report no verified limitations, indicating a robust workforce. However, 4.1% have minor limitations, and 0.6% face significant limitations, underscoring the need for ongoing health assessments. The age distribution of ambulance drivers shows that 50.0% are under 30, indicating a youthful workforce. However, 17.4% are aged 30 to 55, while 63.4% are over 55, highlighting a significant proportion nearing retirement age (Table 10).

### 3.5. Communication Systems and Effectiveness in Coordinated Response

The evaluation of communication infrastructure within emergency medical services reveals significant insights into system readiness and adherence to established protocols, which are vital for gauging the operational effectiveness of these services. Notably, a designated phone number for urgent responses is in place in 59 instances (34.3%), suggesting that just over one-third of the analyzed entities have a direct line for emergencies. Nevertheless, the reliance on various other numbers by 39.5% of the units could potentially complicate the efficiency of call handling during emergencies (Table 11).

The ability to identify incoming calls—a crucial factor in prioritizing emergency responses—is implemented in 45.3% of the units. This feature substantially boosts the responsiveness of services, facilitating the swift identification of repeat or critical calls. In contrast, the absence of this capability in 28.5% of the units might hinder timely responses, necessitating manual verification of calls.

Variability is also evident in the assignment of call reception responsibilities: doctors are directly engaged in this task in only 14.0% of cases, whereas nurses or technicians assume this role in another 14.0% of instances, occasionally requiring consultation with a doctor, as noted in 10.5% of the cases. The most common arrangement involves a mixed model where both nurses and doctors participate, as observed in 35.5% of the responses. This arrangement likely offers a balanced approach, ensuring that skilled medical personnel are involved early in the triage process (Table 11).

Furthermore, protocols for managing calls, essential for the standardization and efficiency of operations, are in place in 54.1% of the units. This demonstrates a predominant compliance with structured communication protocols, which are critical for upholding high standards of service. However, the lack of established protocols in 19.8% of the units may result in inconsistent management of incoming calls. The data underscore both the strengths and potential areas for enhancement in the communication frameworks of emergency medical services. By promoting more standardized call-handling practices and advancing the technological infrastructure for call identification, there could be substantial improvements in service delivery, especially in scenarios characterized by high urgency and stress (Table 11).

Our findings demonstrate that the efficiency of communication systems within EMS has a direct impact on the coordination and execution of emergency responses. Establishing strong communication protocols and infrastructure is crucial for enhancing collaboration between EMS teams and other emergency services during critical incidents.

When examining the capabilities and technological backbone of emergency medical services, several key factors stand out, particularly in the areas of communication tools, response protocols, and direct lines of communication. This analysis delves deeply into the hardware and operational dynamics crucial for swift and efficient disaster management. Regarding that, about 35.5% of services report having operational dictation machines, which play a vital role in documenting interactions accurately and maintaining accountability. However, 9.3% have machines that are not working, potentially hampering effective record-keeping and follow-up on emergency calls. Furthermore, 29.1% of the services lack a dictation machine entirely, which could affect the quality of data retention and retrieval (Table 12).

In 41.3% of cases, phone conversations with patients are recorded, aiding in thorough documentation and review of emergency calls, essential for training and quality control. However, 32.6% of services do not adopt this practice, and for 26.2%, it is not applicable, possibly due to privacy concerns or technical limitations. Moreover, only 8.7% record radio communications, with a significant 65.1% not doing so, which might impact the review and enhancement of dispatch and on-field communication protocols (Table 12).

A mere 12.2% have a direct telephone line to the police, and only 11.6% connect with the Alert and Notification Center, indicating limited direct liaisons with these crucial emergency services. This limitation could delay response times during incidents that require police or centralized alert services. A majority (62.2%) of communications with field teams happen via mobile phones, highlighting reliance on cellular networks for coordination. The combined use of mobile phones and radios is seen in only 8.1% of cases, while exclusive use of radios is minimal at 3.5%, showing a shift towards more accessible and potentially more reliable mobile technology (Table 12).

While 15.7% of ambulances have a radio station, a significant 58.1% do not, which may impede communication during critical transfers or remote interventions. Radio repeaters, vital for extending the range of radio communications, are operational in just 16.9% of cases, with a worrying 57.0% reported as non-operational, highlighting a critical need for improvement to ensure robust communication during emergencies. Only 17.4% of services have devices to power their radio systems during outages, revealing a vulnerability in maintaining communication continuity during infrastructure failures. Direct channels to communicate with police and firefighter–rescuers are exceptionally rare, at just 1.7% each, pointing to a significant gap in establishing dedicated and efficient communication lines with these critical emergency response entities (Table 12).

Tracking reaction times during first-order emergency interventions occurs in 39.5% of cases, a crucial metric for assessing the responsiveness and efficiency of emergency services. However, nearly one-third (34.3%) do not monitor these times, potentially missing out on valuable data that could drive improvements in service delivery. These insights collectively underscore both the strengths and significant areas for improvement in enhancing the effectiveness, speed, and reliability of emergency medical services. Advancing technological infrastructure, ensuring the functionality of communication tools, and setting up solid protocols for direct communication with other emergency services are vital steps towards optimizing emergency response outcomes (Table 12).

### 3.6. Emergency Response Times and Effectiveness in Urgent Interventions

An analysis of response times in different emergency medical service scenarios sheds light on how effectively and swiftly interventions are conducted. By categorizing these times into distinct ranges, we can better understand how well services are delivered across various operational contexts. The data indicate that a significant 20.3% of activation times fall within the 0-to-1 h range, demonstrating a quick response in a fifth of the cases. However, as the activation time lengthens, the percentage of cases decreases, with only 6.4% taking between 1 and 3 h and an even smaller 4.1% extending from 3 to 10 h. Alarmingly, 8.7% of activation times exceed 10 h, raising questions about delays in certain emergency responses and pointing to either complex cases or underlying inefficiencies that merit closer scrutiny (Table 13).

On the other side, reaction times offer another critical measure of responsiveness. In 8.7% of cases, services manage to react within an hour, indicating that immediate action is possible, though not consistently achieved across all services. The largest proportion, 23.3%, falls within the 1-to-10 h range, revealing a wide variation in response timeliness. Reaction times that extend beyond 10 h—5.8% of cases up to 20 h and 1.7% surpassing 20 h—highlight possible operational or logistical challenges (Table 13).

This metric (prehospital intervention time) breaks down the responsiveness from the onset of an emergency to the point of medical intervention. About 15.7% of prehospital interventions occur within 0 to 10 h, reflecting quicker activation periods. The percentage rises to 16.3% for interventions taking 10 to 30 h, suggesting that many interventions fall within this range. Longer intervention times—between 30 and 60 h and those exceeding 60 h—are less common, at 5.2% and 2.3%, respectively, indicating areas where intervention delays are significant. These various timeframes—across activation, reaction, and prehospital intervention—provide a detailed view of the operational dynamics in emergency medical services. The data reveal both the potential for rapid action and areas where delays impact overall emergency response effectiveness. Understanding these dynamics is essential for pinpointing where improvements are needed, enhancing training and resources, and ultimately improving patient outcomes in critical situations such as disasters (Table 13).

The study highlights that optimizing response times is crucial for improving patient outcomes during urgent interventions. Tackling logistical challenges and enhancing resource distribution can greatly reduce delays and boost the overall efficiency of EMS operations in emergencies.

A notable number of institutions have developed written plans or procedures for disaster response, with nearly half (48.8%) confirming their existence. This statistic indicates a moderate level of preparedness, as almost 50% of the surveyed institutions have established and shared their disaster response strategies with their teams. On the flip side, only 5.8% reported having vehicles equipped specifically for mass casualty incidents, pointing to a considerable gap in physical readiness for large-scale emergencies. Similarly, only 9.3% of institutions had triage cards, which are essential for the efficient allocation of resources during such incidents. This limited availability suggests there could be delays and inefficiencies during the initial response phases (Table 13).

In terms of mass casualty response drills, just 14% of institutions conducted these exercises in the past 2 years, with most of them doing so once a year or even less frequently. This lack of regular training could impact the readiness and ability of institutions to effectively manage disaster situations. Furthermore, only 15.7% of institutions participated in joint drills with other emergency services, such as the police, military, and fire departments, over the past 2 years (Table 13). This could hamper coordinated response efforts in real disaster scenarios.

Looking at response times as another key measure of preparedness, only 8.7% of services managed to respond within an hour, showing that while immediate action is sometimes possible, it is not consistently achieved across all services. The largest group, 23.3%, responded within 1 to 10 h, illustrating a wide variation in timeliness. Some response times exceeded 10 h, with 5.8% taking up to 20 h and 1.7% taking even longer, highlighting potential operational or logistical challenges (Table 13).

### 3.7. Training and Preparedness for Disaster (Emergency) Response

Regarding training compliance and needs within an EMS system, results show that 32.0% of newly hired doctors and 30.8% of nursing technicians have undergone specialized emergency medicine training soon after being hired, reflecting strong initial training programs for these essential staff members. However, there are notable gaps, as 41.9% of doctors and 43.0% of nursing technicians reported not receiving this training, highlighting a need to improve training coverage (Table 14). A closer look reveals that 42.4% of EMS personnel have received training at established centers, yet 31.4% have not, and 26.2% are marked as not applicable, indicating potential disparities in training access or inconsistent requirements across the organization. Alarmingly, while a significant 68.0% of respondents recognize the need for more training for all EMS staff, 5.8% do not see this necessity, pointing to a perception gap that could affect service delivery (Table 14).

When asked to prioritize training needs, 57.0% of respondents believe doctors require the most training, compared to only 5.2% for nursing technicians and ambulance drivers. This suggests a focused need for advanced training for physicians who often handle the most complex medical emergencies (Table 14). Specific training needs identified include CPR and trauma management, prioritized by 26.2% of responses, underscoring the importance of these skills in emergency settings. Other areas such as urgent medical conditions, emergency protocols and equipment use, safety and operational training, and specialized medical fields like obstetrics and cardiology also receive significant attention. This indicates a broad spectrum of training needs that align with the diverse challenges faced in emergency medical services (Table 14).

Our analysis underscores the critical role of continuous training and preparedness in improving EMS personnel’s effectiveness in disaster response. Institutions that emphasize training tend to show higher levels of readiness and competence when handling large-scale emergencies, highlighting the importance of regular drills and educational programs.

A significant majority, 57%, emphasized the importance of establishing clear norms for operations, including aspects like equipment, staffing, space, vehicles, and education. This highlights a strong belief in the necessity for standardized and well-defined operational guidelines. Similarly, 47.1% of participants stress the importance of strict adherence to established standards and procedures, underscoring the critical role of regulatory compliance and structured protocols in disaster management (Table 14).

Moreover, continuous education is considered crucial, with 56.4% of respondents acknowledging its importance in keeping EMS personnel updated with the latest medical practices and emergency response techniques. However, opinions differ on the value of physical expansions like new training centers; only 34.3% see this as beneficial, while a larger portion, 39.5%, disagrees, suggesting concerns about the effectiveness of resource allocation for such initiatives. Support for equipment renewal is notably strong, with 61.6% of respondents affirming that modern and efficient equipment is vital for enhancing EMS functionality and service quality (Table 14).

Similarly, 59.3% of respondents favor adding more staff, pointing to a recognized need for additional personnel to meet increasing service demands and ensure prompt emergency responses (Table 14). These perspectives collectively highlight a consensus on the need to improve operational standards, continuous professional development, and resource upgrades to advance the quality and efficiency of emergency medical services. However, the perceived value of expanding training facilities remains a point of contention.

### 3.8. Financial Resources and Administrative Effectiveness in Emergency Medical Services

A significant 69.2% of Emergency Medical Services (EMS) are primarily funded by the National Health Insurance Fund (RFZO), showing a heavy reliance on national health insurance to keep their operations running smoothly. In stark contrast, just 4.1% of EMS do not receive any funding from RFZO, underscoring the fund’s crucial role in supporting EMS activities. On the other hand, municipal or city budgets are a source of funding for 39% of EMS, while 34.3% receive no financial support from their local governments, illustrating differing levels of local government involvement in various areas (Table 15).

When it comes to generating their own revenue or receiving donations, these sources play a smaller role. Only 24.4% and 20.3% of EMS rely on self-generated revenue and donations, respectively. Notably, 48.8% do not depend on their own revenue, and 52.9% do not rely on donations, pointing to potential financial vulnerabilities and challenges in maintaining stable operations. Additionally, 40.7% of healthcare institutions benefit from extra funding from local governments for staffing, which is vital for enhancing their ability to respond effectively to emergencies (Table 15).

In terms of staffing, about half of the EMS units operate with a modest number of 0–5 doctors, indicating that many services manage with minimal medical personnel. This pattern is similar for medical nursing technicians and ambulance drivers, suggesting that numerous EMS units work with limited staff. The data on service length and working conditions reveal that a significant number of doctors and medical technicians/nurses have considerable experience, suggesting good retention rates. However, almost as many lack this longevity, which could indicate staff turnover or the presence of newer team members. Compensation for working unsociable hours is well recognized, with around 69.8% of medical staff receiving payment for night shifts and work on Sundays. This highlights the demanding nature of EMS work schedules and the importance of compensating staff for their night and weekend shifts (Table 15).

The findings indicate that sufficient financial resources are critical to the operational success of EMS. Securing adequate funding for personnel, equipment, and infrastructure is vital for upholding high standards of care and ensuring preparedness during emergencies.

The assessment of the ambulance vehicle fleet shows a diverse range of ages. Notably, 18.6% of the vehicles were manufactured between 2011 and 2015, suggesting that part of the fleet is relatively modern. However, older vehicles from 1989 to 2000 and 2001 to 2005 make up smaller portions, 6.4% and 11.0%, respectively. This highlights a potential need to update older vehicles to maintain reliability and efficiency in emergency responses (Table 16).

When looking at how much these vehicles have been used, we see that 22.1% have travelled between 400,000 and 1,000,000 km, indicating heavy use. This high mileage suggests that maintaining these vehicles could be costly, and their reliability might be compromised. Other mileage ranges also reflect significant usage, underscoring the demanding nature of these vehicles’ operational duties. Regarding the availability of critical medical equipment, the functionality rate is quite high for essential items like EKG machines and biphasic defibrillators at 70.9% and 64.0%, respectively. This suggests that most EMS services are well-prepared to handle cardiac emergencies. However, there is a noticeable gap in more advanced equipment like portable mechanical respirators with CPAP mode, which are functional in only 11.0% of services. This indicates a need for improvement in respiratory support capabilities. Basic emergency equipment, such as cardiopulmonary resuscitation sets and 10-L oxygen bottles, is generally well-stocked, with functionality rates of 61.6% and 68.6%, respectively. This suggests a strong readiness for basic life-saving interventions. However, more specialized equipment, like vacuum mattresses and cervical collars for spinal immobilization, show varied availability at 33.1% and 62.8%, respectively, indicating differences in preparedness for specific emergencies (Table 16).

Communication equipment is another critical area for EMS operations. Fixed radio stations are available in 25% of ambulances, while handheld radios are present in only 9.3% of cases. This highlights a significant opportunity to enhance communication capabilities during emergencies. There are also significant shortages in essential medical supplies, such as thrombolytic medications and emergency cricothyrotomy kits, which are available in only 4.7% and 8.7% of cases, respectively. Similarly, the availability of infusion solution heaters and protective helmets with lamps is extremely limited, at just 0.6% each. These shortages emphasize the challenges EMS faces in being fully equipped. Overall, these findings point to critical needs and gaps within emergency medical services. There’s an urgent requirement for investment in equipment and vehicle updates to boost the effectiveness and responsiveness of EMS operations (Table 16).

## 4. Discussion

This study identified the risks and levels of efficiency within the functioning of the Emergency Medical Service (EMS) under both regular and emergency circumstances, such as disasters. The primary focus of the research was on scientifically predicting and explaining the key factors that influence EMS performance, as well as identifying specific strategies and procedures that could improve system efficiency during mass casualty events, including emergencies and disasters. The research results highlight significant shortcomings within Serbia’s emergency medical services. This is particularly evident in how resources are managed, personnel are trained, and communication protocols are handled during both regular and emergency situations.

The results of the multivariate regression analysis revealed several areas important for the structure and functioning of EMS. It can be said that the overall structure of EMS and the total number of EMS points performed were influenced by working hours and shift patterns. On the other hand, it was found that financial resources and ambulance availability were not significantly associated with performance. These results suggest that the allocation of material resources alone may not optimize EMS outcomes [99]. Instead, it can be said that human resource management, particularly scheduling and shift work, has a greater impact [100]. Accordingly, operational factors such as shift scheduling should be prioritized to improve EMS services [101,102]. In contrast, when it comes to service area coverage, none of the variables were significant. Based on these results, it can be concluded that service coverage is likely influenced by more complex factors such as geographic structure and population distribution [103]. Also, EMS teams based in clinics were a strong positive predictor for the number of doctors in EMS. Finally, it was found that for preparedness in the case of mass casualties, such as disasters, ambulance availability, and financial resources were significant predictors. Therefore, although the models explain a small portion of the variance in EMS organizations, they suggest that operational management and resource allocation are key to EMS performance [99,104,105].

The results of Pearson’s correlation provide insight into how certain variables affect the organization of Emergency Medical Services (EMS). The statistically significant correlation between EMS organizations and the number of emergency medicine specialists, doctors in training, and permanent ambulance drivers suggests that an increase in these key personnel improves the efficiency and operational organization of the system. Specifically, a larger number of specialists and trained doctors enhances the capacity of EMS to respond to complex emergencies, while permanent ambulance drivers contribute to continuous operational functionality. The more EMS teams work in shifts during the day and night, the more organized and flexible the system becomes. This finding is crucial for shift planning and staff distribution, as it allows for a better response to changing demands at different times of the day [106,107].

Additionally, the increase in distance between EMS headquarters and hospitals positively correlates with better organization. These results indicate that as distances grow, the need for a more structured organization increases to ensure timely patient transport [108,109]. The findings highlight the importance of geographic coverage and logistical aspects that influence the quality of services provided. In terms of gender dimensions, the results show that a higher number of male doctors contributes to a structurally better organization. This outcome could be explained by potential traditional employment and leadership patterns [110]. In contrast, the increase in the number of female doctors brings balance and diversity to the roles within EMS.

One of the most interesting findings is the positive impact of the increased number of doctors with verified limited working capacity on the number of EMS points performed. This result indicates that institutions are able to efficiently adapt their staff to include doctors with limited working capacity, thereby maintaining operational capacity and ensuring continuity of services. Such findings underscore the significance of diverse inclusive employment policies and flexible resource management [111]. Additionally, it allows for better utilization of all available resources and retention of highly skilled personnel in the system, despite their physical limitations [112,113]. When considering the analysis of shift work, it is important to emphasize that an increase in the number of teams for day and night shifts significantly improves organization and allows for better resource distribution. This leads to a reduction in response time to emergency calls. On the other hand, this result highlights the need for a continuous increase in the number of teams depending on the time of day to ensure optimal coverage at all times [114].

The research results indicate the existence of diverse organizational structures within EMS in Serbian healthcare institutions. Approximately 46% of health centers have EMSs integrated into specialized departments. Also, such organizations can potentially lead to better patient outcomes, as emergency services become more efficient and organized, which is crucial in different disasters [46,115]. Nevertheless, resource requirements to implement this model are quite substantial and may not be sustainable for all facilities [116].

The research shows that in about 34% of centers, the emergency medical service operates within general medical services. This means that existing medical teams are required to handle emergencies alongside their regular duties. Certainly, such an approach allows for maximum resource utilization, but it also raises the issue of balancing routine patient care with emergency demands, which can worsen the quality of emergency response [117]. This type of institution may also require additional staff training to adequately prepare for emergencies [118,119]. A smaller 15% of institutions have established EMS as separate units dedicated solely to emergencies. This structure can increase the efficiency and effectiveness of response through specialized training and equipment [120]. However, this small percentage of units applying this approach suggests that there are barriers, such as financial and administrative obstacles, preventing its wider implementation [62,121].

When it comes to specialized institutions, such as the Institute for Emergency Medical Services, it was found that they exist in only 3.6% of cases. These institutions are specialized, skilled, and equipped with resources for emergency response. They could be recognized as centers of excellence, providing advanced emergency medical care and serving as a model for best practices [122]. On the other hand, nearly 1.44% of facilities do not have an organized emergency medical system. This indicates a lack of emergency healthcare coverage and the necessity for political interventions and investments to expand emergency services [46].

Emergency transport and other emergency healthcare activities are primarily centralized, with 88.89% of operations conducted at one or two locations. While centralized operations can be efficient due to economies of scale, they limit access to remote areas and can pose challenges for rural and less developed regions [85,123]. In some cases, institutions attempt to improve coverage through limited decentralization (4.44% have 3 to 10 locations, and 1.11% have more than 11 locations). However, further decentralization is required to ensure equal access to emergency services across the country [69]. Centralized operations can also lead to excessive demand during high-need periods, highlighting the need for resource planning to increase capacity and responsiveness [124].

In this latter case, it can be said that ambulances are staffed by a combination of rotating shifts and adopted shift work models, with most operations (55.2%) adopting a shift-based approach. This provides the impression that this method is less disruptive to daily life than rotating shifts and provides continuous 24/7 emergency medical care coverage. With 80.2% of institutions using 12 h shifts, work efficiency and staff well-being are high, although this can lead to fatigue and performance decline over time. Other new models, such as 8 h shifts and flexible schedules, may also reduce burnout rates and increase employee satisfaction [125,126]. It was also found that most shifts (50.6% during the day and 48.3% at night) operate with a single team. This certainly simplifies operations but may lead to problems in the event of a sudden surge in demand or complex emergencies [127]. This may indicate the need for institutions to use multiple teams (16.9% during the day and 16.3% at night) or special configurations (27.9% during the day, 23.2% at night), as these models could be adapted to demand-based needs, which could improve EMS efficiency [53].

The results show that the variability in the composition and deployment of medical transport teams is pronounced, with 43.0% of centers conducting daytime transport operations using a single team. This “lean” system, which emphasizes resource efficiency [128], may have limited capacity for handling complex cases or multiple emergency interventions simultaneously [43]. The teams consist of doctors, medical technicians/nurses, and drivers, ensuring complete patient care but utilizing more human resources (37.8%). Although healthcare facilities cover an average area of 350 km^2^, the lack of highway access in 50.0% of institutions indicates potential problems with the timely arrival of ambulances and the provision of rapid response [129]. Improved accessibility and EMS performance require strategic facility deployment and infrastructure development (including roads and communication systems) [68,130].

The study also highlights several serious weaknesses in strategic preparedness, the most alarming of which is the fact that 57.0% of institutions do not have detailed disaster management plans. This indicates an opportunity for improved planning and coordinated actions to enhance EMS performance [131,132]. Where preparedness plans are lacking, responses are likely to vary and may be less effective in large-scale emergencies or disasters [133,134].

Reported seasonal population changes among 48.8% of respondents further emphasize the need for flexible and adaptive strategies within EMS. Adjusting resources and staffing to account for tourism, migration, or temporary residents is crucial in such situations [56,135]. Additionally, the study identifies several distinctive features in staffing models across Emergency Medical Service (EMS) facilities in Serbia. Specifically, one out of every four facilities has a staffing model that includes three to five doctors, adopting a balanced strategy. Of course, this ensures a balance between cost-effectiveness and operational capacity, with enough medical personnel to handle typical emergency cases [41,136]. On the other hand, problems arise in some facilities where 11.63% of units have only 0–2 doctors. Such limited staffing can cause serious issues in providing timely and comprehensive care, especially in high-demand situations or complex emergencies and disasters [42]. Conversely, facilities with larger team sizes—12 to 15 doctors (11.05%) or more than 15 doctors (8.72%)—suggest the ability to manage more complex cases, likely as a result of strategic investments in areas with higher emergency needs or larger populations [45].

Furthermore, it was found that EMS facilities generally employ minimal numbers of specialists, with 51.74% of institutions employing only 0–2 specialists. This shortage further emphasizes recruitment and retention issues, which may affect the quality of care for high-risk or specialized emergency patients [58]. Facilities with three to five specialists (15.70%) can provide more comprehensive care, but the scarcity of a larger number of specialists points to systemic obstacles, such as funding constraints or insufficient opportunities for specialization [71].

It was also found that training for doctors in emergency medicine is sparse, with 67.44% of institutions reporting only 0–2 doctors in training. This situation presents a significant workforce development opportunity [137], highlighting the need for dedicated training programs to enhance specialization and emergency care capacity [60,138]. The trend toward employing general practitioners (58.14% with 0–2 specialists in general medicine) shows a pragmatic approach to staffing but underscores the need for increased specialization to better address diverse medical emergencies.

Further results show that although the representation of female doctors in EMS clinics is relatively good (e.g., 41.3% have 0–5 male and 30.2% have 0–5 female doctors), the distribution of specialists predominantly shows more men (32.7%) compared to female specialists in the same groups. Certainly, these results indicate a relatively balanced gender representation in smaller teams, but larger teams with female doctors are rarer [139]. On the other hand, although women work in EMS, there remains significant room for growth in their representation, particularly at higher levels of specialized roles [48].

Further results show that many medical institutions have very few or no younger doctors under 30 years old. Around 61.0% of institutions employ only 0 to 1 doctors in this age group, indicating challenges in attracting and retaining young doctors, which could affect workforce sustainability [52]. Conversely, the age profile of doctors aged 30 to 55 shows a more stable mid-career workforce, while the significant proportion of doctors over 55 suggests upcoming retirements, requiring active recruitment and succession planning [140].

The part of the research related to communication infrastructure in Emergency Medical Service (EMS) facilities examined their readiness and protocol adherence, which are crucial for successful disaster management [141]. It was found that having a designated phone number for emergency responses, present in 34.3% of institutions, enables faster responses and improves system efficiency [142]. However, when 39.5% of units use multiple phone numbers, inefficiencies in call handling can lead to delays in emergencies. Therefore, establishing a single direct line for emergencies across all institutions could reduce confusion and improve response times in stressful situations [49]. Additionally, the ability to screen incoming calls is available in 45.3% of institutions, which certainly improves responsiveness by enabling the quick identification of key or repeat calls, a crucial feature for prioritizing emergencies [143]. On the other hand, the absence of this feature in 28.5% of units indicates a gap that could lead to delays, suggesting a need for technological upgrades to support real-time call identification [144].

Further analyses also revealed variability in the duties for receiving calls, with the most common model being a combination of nurses and doctors (35.5%). This balanced approach ensures early medical involvement in the triage process [47]. On the other hand, in 14.0% of cases, either nurses or technicians handle the calls, while doctors perform this task in another 14.0% of cases, showing flexibility depending on facility resources. However, requiring doctor consultations in 10.5% of cases may slow down decision-making [145]. Empowering nurses and technicians with additional training could streamline operations and improve response times [146].

The results show that in 54.1% of units, the existence of protocols for handling calls indicates good performance regarding structured communication frameworks. That is essential for maintaining an optimal service standard in a disaster [5,53,69,144]. On the other side, given the lack of standardized protocols in 19.8%, inconsistent call management and delayed responses to emergencies may occur. This must be improved as soon as possible to avoid more serious problems.

Additionally, promoting standardized call-handling practices and widespread protocol implementation across all facilities are necessary approaches to achieving greater operational consistency and service quality [147]. It should be noted that in the EMS environment, communication devices and response protocols, which are an essential part of disaster management, require technological infrastructure [35,36,148,149]. All of the above is important to ensure proper recording of facts, as it is noted that dictaphones function in 35.5% of services, and they should be used for appropriate documentation and accountability.

On the other hand, the absence of operational devices in 9.3% of services and their complete lack in 29.1% of institutions highlights significant shortcomings in data retention and recall capabilities. In this regard, to ensure adequate record-keeping, post-call analysis requires a mechanism that facilitates proper communication in the field, and addressing these shortcomings is crucial. For these reasons, urgent investment in intervention modalities, along with functional communication tools and technology, is necessary [78].

The study also noted challenges in communication with other emergency services, including limited direct communication channels between emergency departments and the police (only 12.2%) and the Alert and Notification Center (11.6%). It is believed that limiting social scanning can slow down responses in situations where coordination with the police or central alarm services is needed [150]. This is a clear signal indicating the importance of integrated communication systems that enable seamless exchange between various emergency services [151]. It can be emphasized that reliance on mobile phones for field communication in 62.2% of cases reflects a shift towards more accessible and reliable technology in practical operations [152,153].

However, the results show that radio usage was very low (3.5%), and the combination of mobile phones and radios (8.1%) was lower than what could have been achieved if institutions used both. Together, this would lead to greater redundancy and reliability during various disasters. The absence of operational radios in ambulances (58.1%) and non-functional radio repeaters (57.0%) also highlights the need for improvements in radio technology, which could ensure a reliable communication environment during emergency scenarios [154].

It is crucial to note that response times in various EMS environments provide important insights into the efficiency and resilience of these services during disasters [55]. Activation time, defined as the period from receiving an emergency call to the start of an emergency response, is another important system readiness indicator [155]. It was found that only 20.3% of activation times fall within the time frame of 0 to 1 h, indicating significant room for improvement in many cases, but on the other hand, it shows an impressive ability to respond quickly where necessary. This finding suggests that if the environment supports it, services can indeed be deployed rapidly [156].

However, it was also found that longer activation times occur (6.4% between 1 and 3 h, 8.7% over 10 h). This could be explained by the fact that in some units, there are improvised emergency teams that only provide transport. Certainly, and as expected, in a system with such limited resources, administrative delays point to bottlenecks—possibly in logistics, financial issues, or the complexity of the disaster itself [15,16,17]. This is important for reducing response times and overall EMS efficiency [157].

Another important aspect related to responsiveness is reaction time, which measures the period between activation and the arrival of the emergency service on-site [20,150]. It was found that 8.7% of services achieve a reaction time of one hour. The variability in how efficiently different services and scenarios are handled suggests that the largest share of cases, 23.3%, falls within the reaction time range of 1 to 10 h [68,130]. Relatively slower response, where 5.8% of cases take 20 h, and 1.7% take more than 20 h, indicates serious operational or logistical challenges. These delays could be the result of traffic congestion problems [158] geographical constraints, or insufficient staffing during high-demand periods [42,85]. It can be noted that solving these problems requires a systematic approach, from infrastructure improvements and faster resource allocation and deployment to enhanced communication systems for quicker response.

It is very important to mention that the prehospital intervention time, or the interval from the onset of an emergency to medical intervention, provides a complete picture of the performance of Emergency Medical Services (EMS) [159]. According to the research results, it was found that 15.7% of interventions occur within 0 to 10 h, which supports rapid mobilization and reaction. On the other hand, it was found that 16.3% of interventions occur within the range of 10 to 30 h, clearly indicating the need for improvement. Also, exceptionally long intervention times, where 5.2% of cases take between 30 and 60 h, and 2.3% exceed that timeframe, suggest the need for strategic adjustments in EMS operations [69]. These delays may result from complex emergencies, limited resources [160], or coordination problems. All of this together highlights the clear need for continuous evaluation and improvement of EMS processes to reduce intervention time and improve patient outcomes [57].

Training for mass casualty incidents, such as disasters, is crucial for EMS preparedness, especially in disaster preparation [161]. According to the research results, around 48.8% of institutions have written plans or procedures for disaster response, indicating moderate preparedness. However, the fact that only 9.3% of institutions have vehicles for mass casualties and triage cards points to shortcomings in preparedness. Such deficiencies can cause delays and inefficiencies during disasters, emphasizing the need for better planning and resource allocation. Additionally, the results indicating the rarity of mass casualty drills, where only 14% of institutions conducted such drills in the past two years, may point to a lack of regular training [61]. Furthermore, it was found that only 15.7% of agencies participate in joint drills with other emergency services, suggesting barriers to coordinated responses. This underscores the importance of improving cooperation and training for an effective disaster response [162].

Further results show that many newly hired employees in Emergency Medical Services (EMS) start with on-the-job training. Specifically, around 32% of new doctors and 30.8% of medical technicians receive emergency medicine training shortly after being hired, reflecting the enhancement of core team members from day one [163]. However, when examined more closely, gaps remain, as 41.9% of doctors and 43% of medical technicians do not receive this training. This highlights the need for broader training programs to ensure comprehensive coverage for all new hires [163]. Consistent training for all new EMS employees is crucial for maintaining high standards of emergency medical care [157]. On the other hand, around 42.4% of EMS staff receive training at established centers, but 31.4% do not have access, and for 26.2%, the training is deemed inapplicable, indicating differences in access to training or inconsistent requirements throughout the EMS system [80]. It is also important to note that 68% of staff recognize the need for more training, while 5.8% do not. If this issue is not addressed, this difference in perception could affect service quality [61,62]. It was found that training priorities are clear, with 57% of respondents stating that doctors require the most training, compared to 5.2% for medical technicians and ambulance drivers. This highlights the need for advanced training for doctors, who often handle complex emergencies and disasters [164]. Key areas of training include cardiopulmonary resuscitation (CPR) and trauma management (26.2%), which are vital skills for disaster response [60,149,157]. Other important areas include emergency medical conditions, emergency protocols, equipment use, and specialized fields such as obstetrics and cardiology, reflecting the wide range of challenges EMS faces [60,149,157].

A significant majority of respondents (57%) support clear operational standards covering equipment, personnel, space, vehicles, and training. This emphasizes the value of standardized guidelines for improving EMS operations [165]. Similarly, 47.1% emphasize the importance of strict adherence to standards and procedures, highlighting the role of compliance with regulations and structured protocols in disaster management. Continuous education is considered vital by 56.4% of respondents, ensuring that EMS personnel stay up to date with the latest practices and techniques [166].

In contrast, opinions on expanding physical training capacities are divided, with only 34.3% seeing it as beneficial, while 39.5% do not. This raises concerns about the effectiveness and resource allocation for such initiatives [59]. However, 61.6% support equipment renewal, believing that modern and efficient equipment is key to improving EMS functionality. Additionally, 59.3% support increasing staff to meet growing service demands and ensure rapid responses to disasters [119,148].

The Emergency Medical Services (EMS) system largely relies on the National Health Insurance Fund (RFZO), which funds 69.2% of its services, emphasizing its critical role. In contrast, only 39% of EMS services receive support from municipal budgets, and 34.3% receive no funding from local governments, indicating significant regional differences in financial support. All of this together highlights the need for greater municipal involvement to ensure consistent service quality [167]. It was found that self-generated income and donations account for only 24.4% and 20.3% of EMS unit funding, respectively, indicating potential financial vulnerabilities [168]. Of course, such results also emphasize the need for diversified funding sources to maintain stable operations [169]. Many EMS units operate with minimal staffing, with around half employing only 0–5 doctors, and a similar trend is seen in the number of medical technicians and ambulance drivers. These staffing levels could affect the quality of emergency responses and highlight the need for more personnel to meet growing service demands [170].

The workforce includes a combination of experienced and newer employees, reflecting both staff retention and turnover. The results show that compensation for night shifts and weekends is provided, with 69.8% of medical staff receiving additional pay, highlighting the demanding nature of EMS work and the importance of fair compensation. The ambulance fleet includes a modest proportion of modern vehicles (18.6% from the period 2011–2015), while many vehicles are outdated, suggesting an urgent need for upgrades to ensure reliability [77]. High mileage in 22.1% of vehicles indicates heavy usage, which could affect efficiency and reliability.

Most EMS units are equipped with basic tools such as EKG machines (70.9%) and defibrillators (64.0%), but there is a significant lack of advanced equipment such as portable respirators (11.0%). The limited availability of communication tools, such as fixed radio stations (25%) and handheld radio devices (9.3%), highlights the need to improve communication capacities [51]. It was also found that there are significant shortages in critical supplies, such as thrombolytic medications (4.7%) and cricothyrotomy kits (8.7%), indicating gaps in preparedness. This underscores the need for better inventory management to support comprehensive emergency response [44].

By identifying these key areas, the research unequivocally presents a clear strategy and recommendations for improving the functioning of the Emergency Medical Service (EMS). Also, the implementation of such recommendations will undoubtedly lead to better disaster response and improved patient care. Additionally, by identifying the key organizational and operational factors that influence EMS efficiency during disasters, it significantly contributes to the overall improvement in the field of EMS. Based on all findings, the foundation is set for improving EMS management practices, including more efficient management of work hours, shift assignments, resource allocation, and the implementation of formal mass casualty response plans.

The study faced several limitations, which are outlined below: (a) the participant sample was drawn exclusively from healthcare institutions within Serbia, limiting the ability to generalize the findings to other countries or healthcare systems; (b) since the research depends on participants’ self-assessments, there is a possibility of subjectivity in their responses, which could introduce bias into the results; (c) the absence of longitudinal data makes it challenging to monitor changes in the efficiency of the emergency medical response system over time, thus potentially obscuring long-term trends; (d) resource and equipment shortages in certain institutions may have influenced the depth of the data collected, especially in facilities with smaller capacities; (e) differences in emergency preparedness levels across healthcare institutions could make it more difficult to compare results from different regions or organizations; (f) the lack of standardized national protocols might have affected the uniformity and reliability of the collected data, posing difficulties for accurate analysis of the system’s overall efficiency.

## 5. Recommendations

The recommendations in Table 17 are designed to tackle critical areas within Emergency Medical Services (EMS) that need strategic improvements. It can be said that by concentrating on elements like organizational structure, resource allocation, communication systems, response times, training, and financial resources, these strategies lay out a detailed plan for boosting the effectiveness and preparedness of EMS operations.

Each recommendation is evaluated based on factors like duration, feasibility, cost, and priority to ensure that the actions taken are well-suited to the varied needs and challenges faced by EMS units. Through these focused efforts, the aim is to strengthen EMS capabilities, make better use of resources, and ensure a strong response to emergencies.

## 6. Conclusions

The analysis of the Emergency Medical Response System (EMRS) in the Serbian healthcare system reveals numerous deficiencies and serious challenges that require the implementation of urgent short-term and long-term measures for improvement. This study is one of the few in Serbia that comprehensively examines multiple indicators of the efficiency and effectiveness of such a system. The extensive study results point to significant and widespread variations in the organization and functioning of EMRS across the country. It can be assumed that these variations are a result of both historical and economic changes, as well as regional specificities. Although certain segments of the system, such as specialized emergency departments, function efficiently, the organization in many areas is inconsistent, leading to inadequate access to emergency medical assistance in some regions of the country. On the other hand, this diversity underscores the need for standardized procedures and more centralized management to improve coordination and optimize efficiency. Certainly, all identified weaknesses must be addressed in the shortest possible time, and the organization and functioning of the system must be improved.

Besides the mentioned facts, the study identified several key weaknesses that significantly affect the efficiency of the system. Among these, outdated infrastructure, a chronic lack of staff, and inadequate logistics stand out, all of which together slow down response times in emergencies, including various disasters. Although reforms aimed at aligning with European standards have been initiated, their implementation is often limited by a lack of financial resources and capacity. The results also show that many emergency services rely on minimal resources and improvisations, while some units operate with insufficiently trained personnel and outdated equipment. Moreover, dependence on the National Health Insurance Fund (RFZO) as the main source of funding further burdens the system, while local budgets and self-generated revenue are insufficient for the stable development and sustainability of EMRS.

Thus, a critical step in improving the system is conducting targeted resource audits and establishing funds for the procurement of modern equipment for emergency medical services. All of these identified weaknesses become particularly evident under the difficult conditions caused by disasters. Therefore, the introduction of mobile units and strategic partnerships with technology providers can significantly improve the distribution of resources, especially in less developed and rural areas. Additionally, sharing resources among EMRS agencies and increasing intersectoral cooperation would optimize the use of available capacities, resulting in greater efficiency.

A particularly important area that requires attention is the various aspects of communication system organization and functionality. In the study, communication was identified as one of the most critical points in the functioning of EMRS. The results clearly indicate significant gaps in communication systems, including a lack of standardized protocols and limited connections with key services such as the police and firefighting–rescue services. The introduction of digital radio systems, regional communication centers, and artificial intelligence to improve inter-agency communication is recommended as an urgent and essential measure. In addition, training on the use of communication technologies is necessary to ensure that EMRS staff is adequately prepared for complex emergency situations, including disasters.

Conversely, the analysis of response times revealed significant variations compared to international standards, indicating the need for additional infrastructure investments and process optimization to reduce response times and improve patient outcomes. For these reasons, focusing on accelerating responses, training personnel, and expanding emergency medical assistance capacity is key to improving system performance.

In light of these findings, it is clearly recommended to establish new training centers and digital hubs for disaster response, as well as to introduce continuous education and specialized training for emergency personnel. Such training is especially significant in areas such as cardiopulmonary resuscitation (CPR), trauma management, and other specialized medical fields. Through intensified cooperation with international agencies and the integration of simulation-based training, Serbia can increase the preparedness and resilience of its emergency medical response system in disaster situations. The results of this study provide a clear roadmap for policymakers, healthcare administrators, and EMRS personnel to define priorities for strategic interventions, strengthen the system, and achieve alignment with international standards, which would significantly improve the health and safety of Serbian citizens.

The conducted research has unequivocal scientific and societal implications in the area of emergency medical services improvement, as well as in disaster management and response. The scientific implications are reflected in enriching the existing literature in these fields and enhancing the understanding of key factors influencing the efficiency of emergency medical services (work organization, resource distribution, logistical challenges, etc.). A rich repository of data with empirical evidence is created, which can be used for comparison with other countries. Moreover, the study clearly identifies the need for standardization of procedures, protocols, and management within emergency medical services. This creates a foundation for future research on how standardization can contribute to improving response times and reducing regional disparities in access to such services. Additionally, through the use of statistical methods such as Pearson’s correlation, multivariate regression analysis, and chi-square tests, the study provides a methodological framework for further investigation into the organizational and operational aspects of emergency medical services.

Regarding societal implications, the study offers concrete recommendations for healthcare sector reform for policymakers in Serbia and other countries facing similar challenges. Certainly, the results contribute to creating recommendations for increasing the capacity of emergency services and improving response times in medical emergencies, directly affecting the health and safety of the population. The study also highlights the need for better coordination between different sectors, as well as for improving communication systems and logistical capacities. These proposed changes can enhance the resilience of communities in cases of emergencies, disasters, and mass accidents, contributing to the improvement of population safety.

## Figures and Tables

**Table 1:** Number of healthcare institutions (2020). Source: adapted from [93].

Region	n	%
Belgrade Region	57	18.21%
Vojvodina Region	93	29.71%
Western Serbia Region	26	8.31%
Šumadija and Central Serbia Region	55	17.57%
Eastern Serbia Region	31	9.90%
Southern Serbia Region	51	16.29%
Total	313	100
Primary Healthcare Centers	121	38.66%
Institutes for Primary Healthcare	16	5.11%
General Hospitals	10	3.19%
Healthcare Centers	31	9.90%
Special Hospitals	33	10.54%
Tertiary Healthcare Institutions (Clinics, Clinical Centers, University Hospitals, Institutes)	32	10.22%
Institutes for Multilevel Healthcare	34	10.86%
Pharmacies	36	11.50%

**Table 2:** Demographic and institutional characteristics of participants involved in emergency medical response services in Serbia.

Variables	Category	n	%
Function in EMS	Medical personnel	23	13.37
	Leadership positions within a medical institution	140	81.4
	Administrative medical personnel	7	4.07
	Operational medical personnel	2	1.16
Type of Institution EMS	Public health center	122	70.93
	Hospital	11	6.40
	Private healthcare facility	39	22.67
Experience in EMS	Less than 5 years	45	26.16
	5–10 years	85	49.42
	More than 10 years	42	24.42
Gender	Male	95	55.23
	Female	77	44.77
Education Level	High school	30	17.44
	Bachelor’s degree	100	58.14
	Master’s degree	42	24.42
Participation in Training	No participation in training	50	29.07
	Participated in one or more training sessions	122	70.93
Emergency Response Role	First responder	50	29.07
	Coordinator	80	46.51
	Support staff	42	24.42
Mass Casualty Plans/Procedures	Yes, the institution has a plan	110	63.95
	No, the institution does not have a plan	62	36.05
Total		172	100

**Table 3:** Results of a multivariate regression analysis concerning predictors for EMS organization, number of EMS points performed, service area coverage, EMS doctors, and mass casualty plans/procedures.

Predictor Variable	Organization of EMS			Number of EMS Points Performed			Service Area Coverage			EMS Doctors			Plan/Procedures for Mass Casualty		
	*B*	*SE*	*β*	*B*	*SE*	*β*	*B*	*SE*	*β*	*B*	*SE*	*β*	*B*	*SE*	*β*
Organization of working hours	0.03	0.01	0.035 *	−0.032	0.011	−0.037 *	0.029	0.012	0.032	0.038	0.012	0.042	0.041	0.013	0.045
Organization of shift work	0.04	0.012	0.042 *	−0.041	0.013	−0.045 *	0.039	0.014	0.043 *	0.047	0.015	0.048	0.049	0.016	0.051
Organization of work in shifts	0.027	0.009	0.029	−0.028	0.01	−0.031 *	0.026	0.011	0.028	0.034	0.011	0.035	0.036	0.012	0.037
EMS team working only in the clinic	0.065	0.015	0.062	−0.065	0.016	−0.063	0.067	0.017	0.064	0.072	0.018	0.07 *	0.075	0.019	0.073
Teams per day shift for amb. tran.	0.058	0.014	0.058	−0.062	0.015	0.059	0.061	0.016	0.06	0.066	0.017	0.065 *	0.07	0.018	0.069
Teams per night shift for amb. tran.	0.049	0.013	0.053	−0.051	0.014	−0.055	0.05	0.015	0.054	0.055	0.016	0.057	0.059	0.017	0.061
Teams per shift during weekends	0.034	0.01	0.038	−0.036	0.011	−0.039	0.035	0.012	0.037	0.041	0.012	0.043	0.044	0.013	0.046
Financial resources for the healthcare	0.023	0.009	0.029	−0.026	0.01	−0.028	0.025	0.011	0.027	0.031	0.012	0.031	0.033	0.013	0.033
Ambulance vehicles	0.06	0.012	0.06	−0.065	0.013	−0.065	0.067	0.014	0.067	0.072	0.015	0.072 *	0.075	0.016	0.075
Vehicle for mass casualties (disasters)	0.044	0.011	0.041	−0.048	0.012	−0.046	0.047	0.013	0.045	0.052	0.014	0.05	0.055	0.015	0.053
Plan/procedures	0.071	0.016	0.073	−0.071	0.017	−0.074	0.073	0.018	0.075	0.078	0.019	0.078	0.08	0.02	0.08
$R^{2}\;(R_{adj}^{2})$	0.019 (0.006)			0.020 (0.008)			0.027 (0.015)			0.035 (0.022)			0.041 (0.030)		

* *p* ≤ 0.05; *B*: unstandardized (B) coefficients; *SE*: std. error; *β*: standardized (β) coefficients. Note: Note: EMS is organized as a separate institution (Institute for Emergency Medical Services), with multiple dislocated points for EMS, service area coverage of more than 1100 km^2^, higher total number of EMS doctors, the presence of a plan/procedure for mass casualties, shift work for working hours, shifts of 12 h, presence of an EMS team working only in the clinic, adequate financial resources for healthcare, ambulance vehicles functional for daily use, availability of a vehicle for mass casualties, availability and usage of triage tags, participation in exercises for responding to mass casualties, and joint exercises with other first responders have all been coded as 1. All other values have been coded as 0.

**Table 4:** Pearson’s correlation results for the relationship between various structural and operational characteristics of EMS and EMS organization, number of EMS points performed, and service area coverage (n = 172).

Variables		Organization of EMS		Number of EMS Points Performed		Service Area Coverage	
		Sig.	*r*	Sig.	*r*	Sig.	*r*
Structural characteristics	Total number of EMS doctors	0.000 **	−0.340	0.139	0.199	0.190	−0.698
	EMS doctors specialized in emergency medicine	0.000 **	−0.424	0.135	0.213	0.909	0.072
	EMS doctors in emergency medicine training	0.000 **	−0.430	0.139	0.203	0.857	0.112
	EMS doctors specialized in general medicine	0.161	−0.125	0.042	0.702	0.750	0.197
	EMS doctors practicing general medicine	0.001 **	−0.286	0.140	0.199	0.874	−0.126
	Permanent EMS ambulance drivers	0.000 **	−0.344	0.128	0.244	0.905	0.074
	Doctors with verified limited working capacity	0.068	−0.162	0.033*	0.762	0.981	0.015
	Gender distribution of male doctors	0.000 **	−0.346	0.079	0.472	0.995	0.004
	Gender distribution of female doctors	0.009 **	−0.231	0.103	0.343	0.866	0.105
	Male emergency medicine specialists	0.000 **	−0.473	0.072	0.505	0.919	0.063
	Female emergency medicine specialists	0.000 **	−0.338	0.066	0.404	0.435	0.460
Operational characteristics	Day shift teams on weekdays	0.000 **	−0.326	0.115	0.297	0.942	0.046
	Night shift teams on weekdays	0.000 **	−0.409	0.124	0.257	0.992	0.006
	Standby readiness for doctors	0.059	0.164	0.120	0.269	0.892	0.007
	Standby readiness for nurses-techn.	0.088	0.149	0.113	0.299	0.321	0.08
	Maximum diameter of the EMS service area	0.423	0.147	0.366	0.112	0.213	0.09
	Max distance EMC to hospital	0.002 **	0.275	0.132	0.228	0.163	0.837
	Average time spent by medical teams (min)	0.817	−0.020	−0.057	0.597	0.232	0.654
	Average time spent by transport teams (min)	0.732	−0.030	−0.060	0.582	0.338	−0.549

** *p* ≤ 0.01.

**Table 5:** Chi-square test results examine the relationship between different variables and the organization of EMS, employee (EMS) training, and plans/procedures regarding mass casualty.

Variable	Organization of EMS		Employees (EMS) Training		Plan/Procedures Mass Casualty	
	*p*	*X* ^2^	*p*	*X* ^2^		
Conducting EMS activities	0.001 **	190.38	0.001 **	126.64	0.001 **	110.05
Number of points EMS	0.005 **	86.99	0.107	25.68	0.526	16.56
Organization of working hours	0.000 **	194.06	0.002 **	134.01	0.000 **	113.90
Organization of shift work	0.001 **	76.13	0.001 **	34.58	0.055	14.49
Organization of work in shifts	0.006 **	85.40	0.065	23.13	0.000 **	42.14
EMS team working only in the clinic	0.004 **	165.43	0.005 **	147.07	0.060	18.23
Teams per day shift for amb. transport	0.001 **	265.23	0.001 **	154.02	0.000 **	140.43
Teams per night shift for amb. transport	0.000 **	185.06	0.003 **	127.23	0.000 **	126.24
Teams per shift during weekends	0.000 **	223.92	0.001 **	143.07	0.000 **	124.43
Ambulance transport team	0.001 **	142.18	0.056	16.98	0.000 **	133.37
On-call duty, leave the territory	0.003 **	154.18	0.001 **	142.88	0.000 **	123.21
Regular shift workload	0.001 **	140.29	0.006 **	153.06	0.000 **	131.61
Number of doctors in EMC	0.006 **	236.17	0.007 **	160.02	0.000 **	138.07
Systematic medical examinations	0.002 **	126.85	0.285	53.05	0.313	52.24
Verified limited work capacity	0.005 **	115.26	0.320	68.18	0.000 **	148.73
Number of ambulance drivers	0.000 **	211.04	0.160	34.30	0.909	9.128
Separate phone number for amb. transport	0.007 **	129.06	0.000 **	179.29	0.875	35.75
Call identification capability	0.018 *	159.62	0.004 **	175.19	0.453	28.07
Protocol/procedure for receiving calls	0.023 *	116.4	0.000 **	176.43	0.000 **	154.63
Presence of a call recorder	0.003 *	169.14	0.001 **	174.02	0.000 **	150.10
Recording conversations	0.001 **	153.06	0.065	43.65	0.232	46.01
Communication with teams in the field	0.005 **	151.13	0.001 **	179.06	0.000 **	152.96
Presence of radio stations in ambulances	0.001 **	124.43	0.003 **	172.02	0.000 **	148.92
Condition of radio repeaters	0.008 **	134.18	0.204	34.01	0.000 **	18.56
Power supply device for the radio system	0.001 **	123.02	0.001 **	183.02	0.000 **	145.05
Dedicated communication: police	0.005 **	127.01	0.000 **	172.14	0.001 **	124.04
Monitoring reaction time of interventions	0.001 **	174.44	0.000 **	185.93	0.005 **	162.87
Dedicated communication firefighers	0.003 **	119.02	0.002 **	172.14	0.003 **	148.73
Training for emergency medicine doctors	0.004 **	105.01	0.001 **	187.01	0.005 **	139.01
Training for emergency medicine nurses	0.003 **	102.07	0.002 **	175.01	0.001 **	23.08
Financial resources for the healthcare	0.001 **	156.07	0.003 *	165.45	0.000 **	211.76
Ambulance vehicles	0.765	15.06	0.001 **	197.32	0.005 **	175.20
Vehicle for mass casualties (disasters)	0.005 **	130.52	0.001 **	159.67	0.000 **	172.45
Plan/procedure: mass casualties	0.001 **	110.30	0.005 **	149.32	0.000 **	215.65
Triage tags	0.003 **	118.01	0.002 **	150.2	0.045	35.53
Exercises for responding to mass casualties	0.001 **	103.32	0.001 **	149.01	0.000 **	178.34
Joint exercises with other first responders	0.003 **	109.11	0.005 **	139.04	0.000 **	160.14

* *p* ≤ 0.05; ** *p* ≤ 0.01.

**Table 6:** Organizational and operation of emergency medical services (EMSs).

Variables	Category	n	%
Organization of EMS in Facility	No organized emergency medical service	2	1.44
	Special institution—Institute for Emergency Medical Services	5	3.60
	Within a special Emergency Medical Service department of a health center	64	46.04
	Within the general medical service (through regular work and duty of doctors and other health workers)	47	33.81
	Within the general medical service, as a separate organizational unit for emergency medical services	21	15.11
Conducting EMS Activities	From a single location	46	26.7
	Within a healthcare facility	78	45.3
	From multiple dislocated points	13	7.6
Number of points where EMS activities are conducted	From 0 to 2 points	80	88.89
	From 3 to 5 points	4	4.44
	From 6 to 10 points	4	4.44
	From 11 to 50 points	1	1.11
	From 51+ points	1	1.11
Organization of working hours	Shift work	95	55.2
	Rotating shifts	77	44.8
Organization of shift work	In shifts of 12 h	138	80.2
	Other	14	8.1
	In shifts of 8 h	20	11.6
Organization of work in shifts	Day shift—24 h off—night shift—48 h off	33	24.44
	Day shift—24 h off—night shift—72 h off	70	51.85
	Day shift—48 h off—night shift—48 h off	32	23.70
Team configurations during daytime shifts on weekdays	1 team (all variations)	87	50.6
	2 teams (all variations)	29	16.9
	3 or more teams	6	3.5
	Special configurations	48	27.9
Team configurations during nighttime shifts on weekdays	0 teams	2	1.2
	1 team (all variations)	83	48.3
	2 teams (all variations)	28	16.3
	3 or more teams	19	11.0
	Special configurations	40	23.2
Healthcare Management Plan have a team that only works in the clinic	Yes	49	28.5
	N/A	38	22.1
	No	85	49.4
Teams in the clinic during the daytime on weekdays	1 team (including various descriptions)	110	64.0
	2 teams	14	8.1
	3 or more teams (special configurations)	12	7.0
Teams in the clinic during the nighttime on weekdays	0 teams	26	15.1
	1 team (including various descriptions)	124	72.1
	Transport by a team of medical nurse-technician and driver	33	19.2

**Table 7:** Comprehensive overview of emergency medical services (EMS) structure, operations, and geographical coverage.

Variables	Category	n	%
Medical transport teams during daytime shifts on weekdays	No teams reported	9	5.2
	1-team configurations	106	43.0
	2-team configurations	39	14.0
	3-team configurations	7	4.1
	4-team configurations	7	4.1
	5-team configurations	2	1.2
	More than 5-team configurations	3	1.7
Medical transport teams during nighttime shifts on weekdays	No teams reported	34	19.8
	1 team (including all 1-team variations)	126	47.7
	2 teams (including all 2-team variations)	10	5.8
Composition of medical transport teams	Nurse-technician and vehicle driver	67	29.7
	Other	31	13.4
	Vehicle driver	74	33.1
Medical transport team configurations	Standard team (doctor, nurse/technician, driver)	65	37.8
	Driver only or driver with occasional medical staff	29	16.9
	Teams formed based on specific needs	24	14.0
	No specific team required for transport only	17	9.9
	Variable teams depending on patient condition	36	20.9
	Other unspecified configurations	1	0.6
Organization of preparedness for medical teams	Yes	74	43.0
	No	98	57.0
Organization of preparedness for vehicle drivers	Yes	83	48.3
	No	89	51.7
Average holding time of medical teams (in min)	0–10	27	15.7
	11–30	25	14.5
	31–60	27	15.7
	61–120	31	18.0
	121–240	22	12.8
	241 min and above	16	9.3
Average holding time of transport teams (in min)	Under 10	19	11.0
	10–30	26	15.1
	31–60	32	18.6
	61–120	30	17.4
	121–240	19	11.0
	241 min and above	6	3.5
Area covered by health services (HMP)	Less than 100 km^2^	12	7.0
	100–200 km^2^	18	10.5
	200–300 km^2^	18	10.5
	300–400 km^2^	35	20.3
	400–500 km^2^	18	10.5
	500–600 km^2^	14	8.1
	600–700 km^2^	14	8.1
	700–800 km^2^	13	7.6
	800–900 km^2^	10	5.8
	900–1000 km^2^	1	0.6
	1000–1100 km^2^	9	5.2
	Other	8	4.7
The largest diameter of territory covered by health services (HMP)	Under 30 km	32	18.6
	30–60 km	104	60.5
	Over 60 km	37	21.5
Maximum distance from HMP headquarters to corresponding hospital	0–25 km	50	29
	25–50 km	53	31
	50–75 km	28	16
	Over 75 km	41	24
Maximum distance from HMP headquarters to the corresponding tertiary healthcare center	Under 30 km	27	15.7
	30 to 60 km	39	22.7
	60 to 90 km	41	23.8
	Over 90 km	49	28.5
Institutions cover part of the highway	Yes	45	26.2
	N/A	41	23.8
	No	86	50.0
Distances that institutions cover part of a highway	Under 25 km	40	23.3
	25 to 50 km	35	20.3
	50 to 75 km	30	17.4
	Over 75 km	22	12.8
Seasonal variations in the population numbers within the HMP’s jurisdiction	Yes	84	48.8%
	N/A	41	23.8%
	No	47	27.3%
Specific population increases reported during seasonal variations within the HMP’s jurisdiction	Under 1000	70	40.7%
	1000 to 5000	64	37.2%
	5001 to 10,000	18	10.5%
	10,001 to 30,000	33	19.2%
	Over 30,000	7	4.1%
Seasonal population increases reported by institutions	Short-Term (1–3 months)	92	53.5
	Mid-Term (4–5 months)	41	23.8
	Long-Term (6 months)	39	22.7
Reasons for the increase in the population or users of HMP services	Seasonal Tourism and Migration	65	37.8
	Returnees and Temporary Residents	34	19.8
	Local Events and Activities	25	14.5
	Migrants	11	6.4
	Regional Center	5	2.9
	Tourist Center	32	18.6
Regular shift’s workload, beyond the scope of managing urgent care, includes additional activities	Yes	112	65.1
	N/A	41	23.8
	No	19	11.0
Extent of additional activities by staff type	From On-Call Duty	43	25.0
	From Regular Staff	129	75.0
Organization of transport teams during the night shift for urgent medical care	Transport by a team of medical nurse-technician and driver	33	19.2
	Transport by a complete medical team (doctor, nurse-technician, driver)	127	73.8
	Transport by a medical vehicle driver only	12	7.0
	Transport by a team of medical nurse-technician and driver	33	19.2

**Table 8:** Distribution of doctors and specialists in emergency medical services.

Variables	Category	n	%
Doctors in emergency medical services	0–2 doctors	20	11.63
	3–5 doctors	44	25.58
	6–8 doctors	31	18.02
	9–11 doctors	31	18.02
	12–15 doctors	19	11.05
	More than 15 doctors	15	8.72
Specialists in emergency medical services	0–2 doctors	89	51.74
	3–5 doctors	27	15.70
	6–8 doctors	7	4.07
	9–11 doctors	3	1.74
	12–15 doctors	2	1.16
	More than 15 doctors	4	2.33
Doctors in emergency medical services (EMS) specialists who are in training for emergency medicine	0–2 doctors	116	67.44
	3–5 doctors	8	4.65
	6–8 doctors	2	1.16
	9–11 doctors	1	0.58
	12–15 doctors	1	0.58
	More than 15 doctors	0	0.00
Doctors in emergency medical services (EMS) who are specialists in general medicine	0–2 doctors	100	58.14
	3–5 doctors	23	13.37
	6–10 doctors	4	2.33
	More than 10 doctors	1	0.58
General medicine doctors in emergency medical services (EMS)	0–4 doctors	74	43.02
	5–9 doctors	42	24.42
	10–19 doctors	11	6.40
	20 or more doctors	0	0.00
Institutions have other specialities	Yes	94	54.7
	Not Applicable	43	25.0
	No	35	20.3
Medical specialities in institutions	General medicine (general practitioners)	30	17.4
	Specialized medicine (all specialized fields like gynaecology, paediatrics, etc.)	85	49.4
	Diagnostics and lab (radiology, biochemistry, etc.)	40	23.3
	Surgical specialities (surgery-related fields)	10	5.8
	Other specialties (less common specialties)	7	4.1

**Table 9:** Distribution of doctors and nursing staff by gender and specialization.

Variables	Category	n	%
Gender distribution of male doctors	0–5 doctors	71	41.3
	6–10 doctors	27	15.7
	11–20 doctors	21	12.2
	21–30 doctors	4	2.3
	More than 30 doctors	5	2.9
Gender distribution of female doctors	0–5 Doctors	52	30.2
	6–10 Doctors	33	19.2
	11–20 Doctors	30	17.4
	21–30 Doctors	7	4.1
	More than 30 Doctors	6	3.5
Male specialists in emergency medicine	0–2 doctors	107	62.2
	3–5 doctors	16	9.3
	6–10 doctors	5	2.9
	11–15 doctors	1	0.6
	more than 15 doctors	1	0.6
Female specialists in emergency medicine	0–2 doctors	113	65.7
	3–5 doctors	10	5.8
	6–10 doctors	2	1.2
	11–20 doctors	1	0.6
	more than 20 doctors	1	0.6
Male doctors in specialization for emergency medicine	0 doctors	97	56.4
	1–2 doctors	24	14.0
	3–5 doctors	6	3.5
Female doctors in specialization for emergency medicine	0 doctors	93	54.1
	1–2 doctors	28	16.3
	3 or more doctors	5	2.9
Male general medicine specialists	0 doctors	77	44.8
	1–2 doctors	42	24.4
	3 or more doctors	8	4.7
Female general medicine specialists	0 doctors	61	35.5
	1–2 doctors	41	23.8
	3–5 doctors	17	9.9
	6 or more doctors	7	4.1
Male general medicine doctors	0–2 doctors	84	48.8
	3–5 doctors	33	19.2
	6–10 doctors	9	5.2
	more than 10 doctors	1	0.6
Female general medicine doctors	0–2 doctors	50	29.1
	3–5 doctors	35	20.3
	6–10 doctors	24	14.0
	11 or more doctors	17	9.9
Male nursing staff with higher education	0–1	104	60.5
	2–4	17	9.9
	5 or more	5	2.9
Female nursing staff with higher education	0–2	95	55.2
	3–5	16	9.3
	6 or more	16	9.3
Male nursing technicians with secondary education	0–5	76	44.2
	6–10	22	12.8
	11–20	13	7.6
	21 or more	16	9.3
	0–5	76	44.2
Female nursing technicians with secondary education	0–5	35	20.3
	6–10	41	23.8
	11–20	35	20.3
	21–30	11	6.4
	31 or more	5	2.9

**Table 10:** Age distribution and work capability in medical and emergency services.

Variables	Category	n	%
Age structure data for doctors under the age of 30	0–1	105	61.0
	2–5	19	11.0
	6 or more	2	1.2
Doctors aged 30–55	0–5	39	22.7
	6–10	39	22.7
	11–20	48	27.9
	21–30	14	8.1
	31 or more	7	4.1
Doctors over the age of 55	0–5	85	49.4
	6–10	19	11.0
	11 or more	23	13.4
Nursing technicians under the age of 30 with secondary education	0–1	89	51.7
	2–4	27	15.7
	5 or more	10	5.8
Nursing technicians aged 30–55	0–5	29	16.9
	6–10	33	19.2
	11–15	24	14.0
	16–20	11	6.4
	21 or more	30	17.4
Nursing technicians over the age of 55	0–2	60	34.9
	3–5	37	21.5
	6–10	18	10.5
	11 or more	11	6.4
Doctors with verified limited work capacity	No limitation	106	61.6
	Minor limitation	18	10.5
	Significant limitation	3	1.7
Doctors comply with the legal requirement to undergo annual systematic medical examinations	Yes	81	47.1
	No	46	26.7
Medical nurses and technicians comply with the legal requirement for annual systematic examinations	Yes	78	45.3
	No	49	28.5
Ambulance drivers regarding compliance with the legal requirement for annual systematic examinations	Yes	111	64.5
	No	16	9.3
Medical nurses and technicians with verified limited work capacity	No Limitation	92	53.5
	Minor Limitation	31	18.0
	Significant Limitation	4	2.3
Ambulance drivers per vehicle in the Emergency Medical Service (HMP)	0–5	77	44.77
	6–15	34	19.77
	16–30	10	5.81
	31–70	5	2.91
	71+	1	0.58
Ambulance drivers in HMP service (permanent employees)	0–16	115	66.9
	17–33	11	6.4
	34–66	1	0.6
Ambulance drivers in the Emergency Medical Service by contract type (Fixed-term Employees)	0–1	89	51.7
	2–3	26	15.1
	4–10	11	6.4
Ambulance drivers on fixed-term contracts with secondary education	0–1	97	56.4
	2–6	25	14.5
	8–20	5	2.9
Ambulance drivers based on their shifts per month with completed traffic school education	0	115	66.9
	1–3	10	5.8
	10	1	0.6
Ambulance drivers who have undergone special training under the National Emergency Medicine Education Program in the past two years	0	104	60.5
	1–6	16	9.3
	8–71	7	4.1
Male ambulance drivers	0–9	98	57.0
	10–29	24	14.0
	30–69	5	2.9
	70 and above	1	0.6
Female ambulance drivers	0	123	71.5
	1–2	2	1.2
	6	1	0.6
Ambulance drivers under the age of 30	0	86	50.0
	1–2	30	17.4
	3–6	9	5.2
	20	1	0.6
Ambulance drivers aged 30 to 55	0–5	82	47.7
	6–15	33	19.2
	17–33	10	5.8
	50 and above	3	1.7
Ambulance drivers over the age of 55	0–3	109	63.4
	4–10	15	8.7
	12 and above	3	1.7
Ambulance drivers with verified limited work capability	0	119	69.2
	1–3	7	4.1
	6	1	0.6

**Table 11:** Communication channels and call management in emergency services.

Variables	Category	n	%
Phone number to call (from the territory under your HMP jurisdiction) in case of intervention	194	59	34.3%
	Other	68	39.5%
The specific phone number for registering for ambulance transport?	Yes	40	23.3%
	N/A	45	26.2%
	No	87	50.6%
Capability to identify incoming calls	Yes	78	45.3%
	N/A	45	26.2%
	No	49	28.5%
Who receives calls	Doctor	24	14.0%
	Nurse/Technician	24	14.0%
	Nurse/Technician only with doctor consultation	18	10.5%
	Mixed model (nurse, doctor)	61	35.5%
Protocol/procedure for receiving calls	Yes	93	54.1%
	N/A	45	26.2%
	No	34	19.8%

**Table 12:** Technical equipment and recording capabilities.

Variables	Category	n	%
Presence and condition of a dictation machine	Yes, functional	61	35.5%
	Yes, non-functional	16	9.3%
	No	50	29.1%
Phone conversations with patients are recorded on a dictation machine	Yes	71	41.3%
	N/A	45	26.2%
	No	56	32.6%
Radio communications recorded on a dictation machine	Yes	15	8.7%
	N/A	45	26.2%
	No	112	65.1%
Special direct telephone line for communication with the police	Yes	21	12.2%
	N/A	45	26.2%
	No	106	61.6%
Direct line for communication with the Alert and Notification Center	Yes	20	11.6%
	N/A	45	26.2%
	No	107	62.2%
Communication conducted with teams in the field	Both methods	14	8.1%
	Via mobile phone	107	62.2%
	Via radio	6	3.5%
Ambulance vehicles have a radio station	Yes	27	15.7%
	N/A	45	26.2%
	No	100	58.1%
Ambulance Vehicles Without a Radio Station	0–5	64	37.2%
	6–10	11	6.4%
	11–15	1	0.6%
	16–20	24	13.9%
Condition of the radio repeaters	Operational	29	16.9%
	Not Operational	98	57.0%
Device to power the radio communication system in case of a power outage	Yes	30	17.4%
	N/A	45	26.2%
	No	97	56.4%
Special radio communication channel for direct communication with the police	Yes	3	1.7%
	No	124	72.1%
Special radio communication channel for direct communication with firefighters-rescuers	Yes	3	1.7%
	No	124	72.1%
Reaction time monitored during first-order emergency interventions	Yes	68	39.5%
	N/A	45	26.2%
	No	59	34.3%

**Table 13:** Analysis of response times in emergency medical services.

Variables	Category	n	%
Activation times	0 to 1 h	35	20.3
	>1 to 3 h	11	6.4
	>3 to 10 h	7	4.1
	>10 h	15	8.7
Reaction time	0 to 1 h	15	8.7
	>1 to 10 h	40	23.3
	>10 to 20 h	10	5.8
	>20 h	3	1.7
Prehospital intervention time results	0 to 10 h	27	15.7
	10 to 30 h	28	16.3
	30 to 60 h	9	5.2
	More than 60 h	4	2.3
A written plan/procedure known to workers in case of disasters	Yes	84	48.8
	N/A	50	29.1
	No	38	22.1
Vehicle for mass casualty incidents equipped with stretchers and medical supplies?	Yes	10	5.8
	N/A	50	29.1
	No	112	65.1
Availability of triage cards (either in vehicles or bags)	Yes	16	9.3
	N/A	50	29.1
	No	106	61.6
Mass casualty response drills in the last 2 years at your institution	Yes	24	14.0
	N/A	50	29.1
	No	98	57.0
Frequently drills for mass casualty incidentsh	One time per year or less	20	80.0
	Twice a year	4	16.0
	More than twice a year	1	4.0
Joint drills with other emergency services in the last 2 years?	Yes	27	15.7
	N/A	50	29.1
	No	95	55.2

**Table 14:** Training and compliance in emergency medical services.

Variables	Category	n	%
Newly hired employee doctors have undergone special training in emergency medicine	Yes	55	32.0
	No	72	41.9
Newly hired employee nursing technicians undergone special training in emergency medicine	Yes	53	30.8
	No	74	43.0
Employees in EMS service undergone training at any of the existing training centers	Yes	73	42.4
	N/A	45	26.2
	No	54	31.4
Additional training is necessary for all employees in the EMS	Yes	117	68.0
	N/A	45	26.2
	No	10	5.8
Importance of who needs training the most	Doctor	98	57.0
	Nursing Technician	9	5.2
	Ambulance Driver	9	5.2
Categories of training needs	CPR and Trauma Management (Cardiopulmonary resuscitation, trauma management, polytrauma handling)	45	26.2%
	Urgent Medical Conditions (Emergency response, urgent medical and pediatric care)	35	20.3%
	Emergency Protocols and Equipment (Equipment use, triage, protocols, communication)	32	18.6%
	Safety and Operational Training (Safety protocols, personal safety, psychological support)	30	17.4%
	Specialized Medical Training (Obstetrics, toxicology, neurology, cardiology)	30	17.4%
Specification of norms for operations (equipment, staff, space, vehicles, education, etc.) as key area for enhancing EMC services	Yes	98	57.0
	N/A	45	26.2
	No	29	16.9
Implementation and adherence to standards and procedures as key area for enhancing EMC services	Yes	81	47.1
	N/A	45	26.2
	No	46	26.7
Continuous education as key area for enhancing EMC services	Yes	97	56.4
	N/A	45	26.2
	No	30	17.4
Establishing new training centers as key area for enhancing EMC services	Yes	59	34.3
	N/A	45	26.2
	No	68	39.5
Equipment renewal as key area for enhancing EMC services	Yes	106	61.6
	N/A	45	26.2
	No	21	12.2
Additional Staff as key area for enhancing EMC services	Yes	102	59.3
	N/A	45	26.2
	No	25	14.5

**Table 15:** Funding sources and staffing in emergency medical services (EMSs).

Variables	Category	n	%
National health insurance fund (RFZO) resources: source of funding EMS	Yes	119	69.2
	N/A	46	26.7
	No	7	4.1
Municipal/City Budget Resources: source of funding EMS	Yes	67	39.0
	N/A	46	26.7
	No	59	34.3
Own Revenue: source of funding EMS	Yes	42	24.4
	N/A	46	26.7
	No	84	48.8
Donations: source of funding EMS	Yes	35	20.3
	N/A	46	26.7
	No	91	52.9
Healthcare institution receive additional financial resources from local government to employ additional staff	Yes	70	40.7
	N/A	47	27.3
	No	55	32.0
Doctors in Emergency Medical Services	0–5	86	50.0%
	6–10	14	8.1%
	11–15	8	4.7%
	16–20	8	4.7%
	21+	9	5.2%
Medical Nursing Technicians in Emergency Medical Services	0–5	83	48.3%
	6–10	18	10.5%
	11–15	11	6.4%
	16–20	8	4.7%
	21+	5	2.9%
Ambulance Drivers in Emergency Medical Services	0–5	84	48.8%
	6–10	16	9.3%
	11–20	15	8.7%
	21–30	7	4.1%
	30+	3	1.7%
Doctors in Emergency Medical Services have credited service years	Yes	56	32.6
	No	66	38.4
	Undecided	3	1.7
Doctors in Emergency Medical Services have paid night shifts?	Yes	120	69.8
	No	2	1.2
	Undecided	3	1.7
Doctors in Emergency Medical Services have paid work on Sundays?	Yes	120	69.8
	No	5	2.9
Medical technicians/nurses in Emergency Medical Services and ambulance transport have credited service years?	Yes	57	33.1
	No	66	38.4
	Undecided	2	1.2
Medical technicians/nurses in Emergency Medical Services and ambulance transport have paid night shifts?	Yes	120	69.8
	No	2	1.2
Medical technicians/nurses in Emergency Medical Services and ambulance transport have paid work on Sundays	Yes	120	69.8
	No	5	2.9

**Table 16:** Availability of medical equipment and emergency personnel in EMS.

Variables	Category	n	%
Ambulance vehicles by year of manufacture	1989–2000	11	8.15
	2001–2005	19	14.07
	2006–2010	21	15.56
	2011–2015	32	23.70
	2016–2018	20	14.81
	2019–2023	32	23.70
Medical vehicles—number of kilometres travelled	0–57,200	21	20.2
	57,200–125,354	19	18.3
	125,354–285,564	20	19.2
	285,564–400,000	21	20.2
	400,000–1,000,000	23	22.1
The presence of radio stations in medical vehicles	Yes	38	22.0
	No	70	40.7
Functionality of EKG machines for activities within the healthcare service	Does not exist	2	1.2
	Exists	122	70.9
Biphasic defibrillators with monitors for activities within the healthcare service	Does not exist	13	7.6
	Exists	110	64.0
Functionality of portable aspirators for activities within the healthcare service	Does not exist	17	9.9
	Exists	106	61.6
Portable mechanical respirator with oxygen tank functionality in HMP activities	Does not exist	59	34.3
	Exists	64	37.2
Functionality of portable mechanical respirators with oxygen tanks that have the CPAP mode	Does not exist	104	60.5
	Exists	19	11.0
Availability of cardiopulmonary resuscitation sets	Does not exist	17	9.9
	Exists	106	61.6
Availability of 10-litre oxygen bottles for activities within the healthcare service	Does not exist	5	2.9
	Exists	118	68.6
Vacuum mattresses for activities within the healthcare service	Does not exist	66	38.4
	Exists	57	33.1
Cervical collars for spinal immobilization	Does not exist	15	8.7
	Exists	108	62.8
Kramer splints for activities within the healthcare service	Does not exist	39	22.7
	Exists	84	48.8
Infusion solution heater functionality in HMP activities	Does not exist	122	70.9
	Exists	1	0.6
Medications for thrombolytic therapy	Does not exist	115	66.9
	Exists	8	4.7
Emergency cricothyrotomy kits	Does not exist	108	62.8
	Exists	15	8.7
Availability of childbirth kits	Does not exist	38	22.1
	Exists	85	49.4
Protective helmets with lamps availability in HMP activities	Does not exist	122	70.9
	Exists	1	0.6
Fixed radio station availability in ambulance	Does not exist	80	46.5
	Exists	43	25.0
Handheld radio availability	Does not exist	107	62.2
	Exists	16	9.3
Ultrasound device availability	Does not exist	112	65.1
	Exists	11	6.4

**Table 17:** Strategic recommendations for enhancing emergency medical services: addressing structural, resource, and operational challenges.

Aspect	Recommendations	Term	Feasibility	Cost	Priority
Organizational Structure and Risk Management	Standardize risk assessments across all EMS units	Short	High	Low	High
	Introduce dynamic updating protocols for emergency response strategies	Short	High	Medium	High
	Establish a centralized authority for EMS management	Long	Medium	Medium	High
	Integrate new technology platforms for real-time risk management	Long	Low	High	High
	Develop inter-agency agreements for risk management best practices	Long	Medium	Medium	Medium
Resource Allocation and Efficacy	Conduct targeted resource audits in high-demand locations	Short	High	Low	High
	Deploy mobile resource units in underserved areas	Short	Medium	High	High
	Establish a fund for state-of-the-art EMS equipment	Long	Medium	High	Medium
	Develop partnerships with technology providers	Long	Low	High	Medium
	Implement a resource-sharing protocol among EMS agencies	Long	High	Low	Low
Communication Systems and Efficacy	Upgrade to digital radio systems and secure networks	Short	High	Medium	High
	Establish regional communication centers	Short	Medium	High	High
	Create redundant communication channels	Long	Medium	High	High
	Launch training programs for communication technologies	Long	Medium	Medium	Medium
	Invest in AI-driven communication tools.	Long	Low	High	Medium
Emergency Response Times and Efficacy	Enhance GPS and dispatch technologies	Short	High	Medium	High
	Develop rapid deployment strategies	Short	Medium	High	High
	Invest in infrastructure improvements at EMS stations	Long	Medium	High	High
	Expand the network of emergency medical facilities	Long	Low	High	Medium
	Implement performance tracking for response times	Long	High	Medium	Medium
Training and Preparedness for Disaster Response	Increase frequency and complexity of disaster response simulations	Short	High	Medium	High
	Develop specialized units for specific disaster scenarios	Short	High	Medium	High
	Collaborate with international disaster response agencies	Long	Medium	High	Medium
	Create a digital training hub for disaster response	Long	Medium	Medium	High
	Mandate disaster preparedness certifications	Long	High	Medium	High
Financial Resources and Administrative Efficacy	Optimize financial planning for high-priority needs	Short	High	Low	High
	Streamline administrative processes to reduce overhead	Short	High	Low	High
	Develop strategic financial partnerships	Long	Medium	Low	Medium
	Use big data analytics for predictive funding needs	Long	Medium	Medium	Low
	Lobby for increased governmental and international funding	Long	Low	Low	High

## Data Availability

Data are contained within the article.

## Appendix A. Questionnaire About Risk and Effectiveness Analysis of Emergency Medical Response Systems in Serbian Healthcare: A Comprehensive Survey on Disaster Emergency Preparedness

**ORGANIZATIONAL STRUCTURE AND RISK MANAGEMENT OF EMERGENCY MEDICAL SERVICES (EMS)**

1. **How is emergency medical service organized in your institution?**
  ○ No organized emergency medical service
  ○ Within the general medicine department (through regular work and on-call duties of doctors and other healthcare workers)
  ○ Within the general medicine department as a separate EMS organizational unit
  ○ Within a separate EMS department of the health center
  ○ Separate institution—Institute for Emergency Medical Services
2. **Is EMS performed from:**
  ○ A single location → Proceed to Question 4
  ○ Multiple dislocated points
  ○ Within the health center
3. **How many points perform EMS?**
  ○ Enter the number of points: _______
4. **What is the work time organization?**
  ○ Shift work
  ○ Rotation work → Proceed to Question 6
5. **What is the organization of shift work?**
  ○ Shifts of 12 h
  ○ Shifts of 8 h
  ○ Other, specify: ______________
6. **What is the organization of rotation work?**
  ○ Day shift—24 h off—night shift—72 h off
  ○ Day shift—48 h off—night shift—48 h off
  ○ Day shift—24 h off—night shift—48 h off
  ○ Other, specify: ______________
7. **Specify the number of teams per shift (consisting of: doctor, nurse-technician, ambulance driver) working:**
  ○ On weekdays: Day shift: _______ Night shift: _______
  ○ During weekends and holidays: Day shift: _______ Night shift: _______
8. **Does EMS have a team working exclusively in the clinic (doctor, nurse/technician)?**
  ○ No
  ○ Yes
9. **Specify the number of teams in the clinic (consisting of: doctor, nurse-technician):**
  ○ On weekdays: Day shift: _______ Night shift: _______
  ○ During weekends and holidays: Day shift: _______ Night shift: _______
10. **How many teams are available for ambulance transport per shift?**
  ○ On weekdays: Day shift: _______ Night shift: _______
  ○ During weekends and holidays: Day shift: _______ Night shift: _______
11. **Who forms the team for ambulance transport?**
  ○ Nurse-technician and ambulance driver
  ○ Ambulance driver
  ○ Specify other: ______________
12. **Is there an on-call duty organized in case the team needs to leave the territory for which it is responsible?**
  ○ No for doctors
  ○ Yes for doctors
  ○ No for nurse-technicians
  ○ Yes for nurse-technicians
  ○ No for ambulance drivers
  ○ Yes for ambulance drivers
13. **What is the average time (in minutes) that the medical team stays at higher-level centers?**
  ○ _______ minutes
14. **What is the average time (in minutes) that the transport team stays at higher-level centers?**
  ○ _______ minutes
15. **Provide data on the territory where EMS services are provided:**
  ○ (a) Area of the territory:
    ▪ Less than 100 km^2^
    ▪ 100–200 km^2^
    ▪ 200–300 km^2^
    ▪ 300–400 km^2^
    ▪ 400–500 km^2^
    ▪ 500–600 km^2^
    ▪ 600–700 km^2^
    ▪ 700–800 km^2^
    ▪ 800–900 km^2^
    ▪ 900–1000 km^2^
    ▪ 1000–1100 km^2^
    ▪ More than 1100 km^2^ (specify _______ km^2^)
  ○ (b) The widest diameter (the greatest distance between two end points) of the territory for which EMS is responsible:
    ▪ 10 km
    ▪ 15 km
    ▪ 20 km
    ▪ 25 km
    ▪ 30 km
    ▪ 35 km
    ▪ 40 km
    ▪ 45 km
    ▪ 50 km
    ▪ 55 km
    ▪ 60 km
    ▪ 65 km
    ▪ More than 65 km (specify _______ km)
  ○ (c) The greatest distance from the EMS headquarters to the competent hospital (secondary level of health care):
    ▪ Up to 10 km
    ▪ Up to 20 km
    ▪ Up to 30 km
    ▪ Up to 40 km
    ▪ Up to 50 km
    ▪ 60 km
    ▪ More than 60 km (specify _______ km)
  ○ (d) The greatest distance from the EMS headquarters to the competent center (tertiary level of health care):
    ▪ Up to 10 km
    ▪ Up to 20 km
    ▪ Up to 30 km
    ▪ Up to 40 km
    ▪ Up to 50 km
    ▪ 60 km
    ▪ Up to 70 km
    ▪ Up to 80 km
    ▪ Up to 90 km
    ▪ Up to 100 km
    ▪ Up to 110 km
    ▪ Up to 120 km
    ▪ Up to 130 km
    ▪ Up to 140 km
    ▪ Up to 150 km
    ▪ More than 150 km (specify _______ km)
16. **Does the institution cover part of the highway?**
  ○ No
  ○ Yes
17. **If yes, specify the length covered: ______________**
18. **Specify the official number of inhabitants for the territory for which EMS is responsible according to the latest data from the Republic Institute for Statistics:**
  ○ _______ inhabitants
19. **Are there seasonal variations in the number of inhabitants on the EMS responsible territory annually?**
  ○ No
  ○ Yes
20. **If yes, specify:**
  ○ For which the largest number of inhabitants increased: _______
  ○ The period for which the seasonal variation of the increase:
    ▪ 1 month
    ▪ 2 months
    ▪ 3 months
    ▪ 4 months
    ▪ 5 months
    ▪ 6 months
    ▪ Other: ______________
21. **Select the reason for the increase in the number of inhabitants/users of EMS services:**
  ○ Tourist center
  ○ Regional center
  ○ Migrants
  ○ Other: ______________
22. **Specify the data source by which the number of inhabitants of the city/municipality is increased/decreased: ______________**
23. **Does the volume of work from the regular shift composition, besides responsibilities from the domain of urgent conditions, include additional activities?**
  ○ No
  ○ Yes
24. **If yes, what are they?**
  ○ House visits from the field service domain (therapy, dressing)
  ○ The job of a medical doctor to professionally determine the time and cause of death outside the health institution and issue a death certificate
  ○ Both mentioned options
  ○ Other (specify): ______________
25. **Are the activities mentioned in the previous question performed from:**
  ○ Regular composition
  ○ On-call duty
26. **During the night shift, in case of the need to transport a patient to a higher-level healthcare institution for urgent care:**
  ○ The entire medical team (doctor, nurse-technician, and ambulance driver) performs the transport
  ○ The team consisting of nurse-technician and ambulance driver performs the transport
  ○ The ambulance driver performs the transport
27. **If the entire medical team accompanies the patient:**
  ○ The team comes from home (on-call duty)
  ○ The doctor and nurse-technician from the clinic accompany the patient
  ○ One of the teams from the shift accompanies the patient

**RESOURCE ALLOCATION (STAFFING) AND EFFECTIVENESS IN EMERGENCY PREPAREDNESS**

1. **State the number of doctors in EMS:**

**Doctors**	**Number**	**Total**
Emergency medicine specialists		
In specialization for emergency medicine		
General medicine specialists		
General medicine		
Other specialties (specify):		

2. **Gender and age structure:**
  1. Gender structure of the total number of doctors:
    ▪ Male: _____
    ▪ Female: _____
  2. Age structure of the total number of doctors (expressed in years, enter the number of persons in squares):
    ▪ Up to 30: ____
    ▪ From 30–55: ____
    ▪ Over 55: ____
  3. Gender structure of emergency medicine specialists:
    ▪ Male: _____
    ▪ Female: _____
  4. Age structure of emergency medicine specialists (expressed in years, enter the number of persons in squares):
    ▪ Up to 30: ____
    ▪ From 30–55: ____
    ▪ Over 55: ____
  5. Gender structure of doctors in emergency medicine specialization:
    ▪ Male: _____
    ▪ Female: _____
  6. Age structure of doctors in emergency medicine specialization (expressed in years, enter the number of persons in squares):
    ▪ Up to 30: ____
    ▪ From 30–55: ____
    ▪ Over 55: ____
  7. Gender structure of general medicine specialists:
    ▪ Male: _____
    ▪ Female: _____
  8. Age structure of general medicine specialists (expressed in years, enter the number of persons in squares):
    ▪ Up to 30: ____
    ▪ From 30–55: ____
    ▪ Over 55: ____
  9. Gender structure of general medicine doctors:
    ▪ Male: _____
    ▪ Female: _____
  10. Age structure of general medicine doctors (expressed in years, enter the number of persons in squares):
    ▪ Up to 30: ____
    ▪ From 30–55: ____
    ▪ Over 55: ____
  11. Gender structure of doctors with other specialties:
    ▪ Male: _____
    ▪ Female: _____
  12. Age structure of doctors with other specialties (expressed in years, enter the number of persons in squares):
    ▪ Up to 30: ____
    ▪ From 30–55: ____
    ▪ Over 55: ____
3. **State the total number of doctors with verified limited work capacity:**
  1. _______________________
4. **Are annual systematic examinations performed according to legal obligations?**
  1. For doctors:
    ▪ No
    ▪ Yes
  2. For nurse-technicians:
    ▪ No
    ▪ Yes
  3. For ambulance drivers:
    ▪ No
    ▪ Yes
5. **How many doctors over the age of 55 have invoked the collective agreement and signed an annex stating that after 55 years they have the right not to work with the field team?**
  1. _______________________
6. **State the number of nurse-technicians in EMS:**
  **Nurse-Technicians**	**Number**	**Total**
  With higher/university education		
  With secondary education		
  1. Gender structure of nurse-technicians with higher/university education:
    ▪ Male: _____
    ▪ Female: _____
  2. Age structure of nurse-technicians with higher/university education (expressed in years, enter the number of persons in squares):
    ▪ Up to 30: ____
    ▪ From 30–55: ____
    ▪ Over 55: ____
  3. Gender structure of nurse-technicians with secondary medical education:
    ▪ Male: _____
    ▪ Female: _____
  4. Age structure of nurse-technicians with secondary medical education (expressed in years, enter the number of persons in squares):
    ▪ Up to 30: ____
    ▪ From 30–55: ____
    ▪ Over 55: ____
7. **State the number of nurse-technicians with verified limited work capacity:**
  1. _______________________
8. **State the number of ambulance drivers in the EMS service:**

**Ambulance Drivers**	**Number**	**Total**
Permanently employed		
Employed for a fixed term		
With 2nd level of education		
With 3rd level of education		
With 4th level of education		
With completed traffic school (3rd level)		
With completed traffic school (4th level)		
With completed traffic school (5th level)		

9. **How many drivers have undergone special training according to the National Education Program in Emergency Medicine for Ambulance Drivers in the last 2 years?**
  1. _______________________
  2. Gender structure of the total number of ambulance drivers:
    ▪ Male: _____
    ▪ Female: _____
  3. Age structure of ambulance drivers (expressed in years, enter the number of persons in squares):
    ▪ Up to 30: ____
    ▪ From 30–55: ____
    ▪ From 55–65: ____
    ▪ Over 65: ____
10. **State the number of ambulance drivers with verified limited work capacity:**
  1. _______________________

**COMMUNICATION SYSTEMS AND EFFECTIVENESS IN COORDINATED RESPONSE**

1. **Which phone number should be called (from the territory for which your EMS is responsible) in case of intervention if the call is made from a mobile phone to reach your EMS?**
  ○ 194
  ○ Or area code: _____ phone number: ______________
2. **Which phone number should be called from the territory for which your EMS is responsible in case of intervention if the call is made from a landline phone to reach your EMS?**
  ○ 194
  ○ Or area code: _____ phone number: ______________
3. **Is there a separate phone number for reporting ambulance transport?**
  ○ No
  ○ Yes

If yes, enter the area code: _____ and phone number: ______________

4. **Is there an option to identify an incoming call?**
  ○ No
  ○ Yes
5. **Who receives the calls?**
  ○ Doctor
  ○ Nurse/technician
  ○ Nurse/technician only in consultation with a doctor
  ○ Mixed model (nurse and doctor)
6. **Is there a protocol/procedure for receiving calls?**
  ○ No
  ○ Yes
7. **Is there a recorder?**
  ○ No
  ○ Yes, but not working
  ○ Yes, working
8. **Are phone conversations with patients recorded on the recorder?**
  ○ No
  ○ Yes
9. **Are conversations via radio recorded on the recorder?**
  ○ No
  ○ Yes
10. **Is there a separate direct phone line for communication with the police?**
  ○ No
  ○ Yes
11. **Is there a direct line for communication with the Alert and Notification Center?**
  ○ No
  ○ Yes
12. **How is communication with teams in the field conducted?**
  ○ By radio
  ○ By mobile phone
  ○ Both
13. **Do all ambulances have a radio station?**
  ○ No
  ○ Yes
14. **How many ambulances, out of the total number, do not have a radio station?**
  ○ Enter percentage: _______%
15. **What is the condition of the radio repeaters?**
  ○ Working
  ○ Not working
16. **Is there a power supply device for the radio system in case of a power outage?**
  ○ No
  ○ Yes
17. **Is there a special radio channel for direct communication with:**
  ○ Police: No/Yes
  ○ Firefighters-rescuers: No/Yes

**REACTION TIME FOR FIRST ORDER EMERGENCIES**

1. **Is reaction time monitored during first-order emergency interventions?**
  ○ No
  ○ Yes
2. **If yes, provide results for the year 2023:**
  ○ Activation time: _______
  ○ Reaction time: _______
  ○ Pre-hospital intervention time: _______

**TRAINING (EDUCATION) AND PREPAREDNESS FOR DISASTER RESPONSE**

1. **Does every new employee undergo special training in the field of emergency medicine according to their job description before independent work in the last 2 years:**
  ○ Doctor: No/Yes
  ○ Nurse-technician: No/Yes
  ○ Ambulance driver: No/Yes
2. **Have the employees in your EMS service undergone training in any of the existing training centers?**
  ○ No
  ○ Yes
3. **Do you consider additional training necessary for all employees in the Emergency Medical Services?**
4. **If yes, select the importance on a scale from 1–3 (1-most needed, 2-medium needed, 3-least needed) for whom the training is most necessary:**
  ○ Doctor: ____
  ○ Nurse-technician: ____
  ○ Ambulance driver: ____
5. **Specify the areas where you believe there is the greatest need for employee education: ______________**
6. **Select the areas you think would most affect the improvement of work within the EMS services (multiple answers possible):**
  ○ Specification of standards for EMS work (equipment, staff, space, vehicles, education, etc.)
  ○ Introduction and adherence to standards and procedures
  ○ Continuous education
  ○ Establishment of new training centers
  ○ Equipment renewal
  ○ Additional staff
  ○ Other (specify): ______________

**ADDITIONAL FUNDING EMERGENCY MEDICAL SERVICES (EMS)**

1. **From which sources is the funding of EMS services carried out (multiple answers possible):**
  ○ From RFZO funds
  ○ From the city/municipality budget
  ○ Own funds of the health institution
  ○ Donations
  ○ Other (specify): ______________
2. **Does the healthcare institution receive additional financial resources from local government for the employment of additional staff?**
  ○ No
  ○ Yes
3. **If yes, specify the number of staff for the emergency medical services by structure:**
  ○ Doctor: _______
  ○ Nurse-technician: _______
  ○ Ambulance driver: _______
    **Do EMS doctors have:**
      ○ Beneficial work experience: No/Yes/Partially
      ○ Paid night work: No/Yes/Partially
      ○ Paid Sunday work: No/Yes/Partially
    **Do EMS and ambulance transport nurse-technicians have:**
      ○ Beneficial work experience: No/Yes/Partially
      ○ Paid night work: No/Yes/Partially
      ○ Paid Sunday work: No/Yes/Partially
    **Do EMS and ambulance transport drivers have:**
      ○ Beneficial work experience: No/Yes/Partially
      ○ Paid night work: No/Yes/Partially
      ○ Paid Sunday work: No/Yes/Partially

**AMBULANCE VEHICLES AND EQUIPMENT**

1. **Ambulance Vehicles:**

**No.**	**Vehicle Type**	**Year of Manufacture**	**Mileage (km)**	**Drive Wheels**	**Radio Station Installed**	**Air Conditioning**	**Functional for Daily Use**	**If not, Specify Reason**
1.	A-van			A-front				
2.	B-van (8 + 1 seats)			B-rear				
3.	C-station wagon			C-all four				
4.	D-passenger car							
5.	J-jeep							

2. **Is the ambulance vehicle functional for everyday work?**
  ○ No
  ○ Yes
3. **The ambulance vehicle was purchased:**
  ○ From the budget of the Government of Serbia/MZ (specify number): ______________
  ○ From the city/municipality budget (specify number): ______________
  ○ Foreign donation (specify number): ______________
  ○ Other: ______________
4. **List the functional equipment for EMS activities:**

**Equipment**	**Present**	**Not Present**	**Need for New (Specify Number)**
EKG device	Yes/No	Yes/No	_______
Biphasic defibrillator with monitor	Yes/No	Yes/No	_______
Biphasic defibrillator with monitor and transcutaneous pacemaker	Yes/No	Yes/No	_______
Biphasic defibrillator with monitor, transcutaneous pacemaker, and capnography option	Yes/No	Yes/No	_______
Aspirator—portable	Yes/No	Yes/No	_______
Portable mechanical respirator with oxygen cylinder	Yes/No	Yes/No	_______
Portable mechanical respirator with oxygen cylinder with CPAP mode option	Yes/No	Yes/No	_______
Set for cardiopulmonary resuscitation (laryngoscope with at least 3 blades of different sizes, endotracheal tubes min. 5 different sizes, self-expanding resuscitation bag, oronasal masks min. 3 different sizes, oropharyngeal tubes min. 3 different sizes)	Yes/No	Yes/No	_______
10-L oxygen cylinder	Yes/No	Yes/No	_______
Portable oxygen cylinder	Yes/No	Yes/No	_______
Trauma care set (bandaging material, straight and curved forceps, Esmarch’s bandage of greater length, scissors for cutting clothes)	Yes/No	Yes/No	_______
Vacuum mattress	Yes/No	Yes/No	_______
Vacuum splints	Yes/No	Yes/No	_______
Collars for immobilization of the cervical spine	Yes/No	Yes/No	_______
Kramer splints	Yes/No	Yes/No	_______
Vest for immobilization and extraction of the injured (KED)	Yes/No	Yes/No	_______
Long spinal board with head immobilizers and body fastening straps	Yes/No	Yes/No	_______
Longitudinally collapsible scoop stretcher for spinal injuries and polytrauma (“Ferno stretcher”)	Yes/No	Yes/No	_______
Infusion solution warmer	Yes/No	Yes/No	_______
Refrigerator for cooling infusion solutions	Yes/No	Yes/No	_______
Transport refrigerator for therapeutic hypothermia equipment	Yes/No	Yes/No	_______
Thrombolytic therapy medications (Streptokinase/Metalyse/Actilyse)	Yes/No	Yes/No	_______
Urgent conicotomy set	Yes/No	Yes/No	_______
Set for intraosseous medication administration	Yes/No	Yes/No	_______
Birth set	Yes/No	Yes/No	_______
Pulse oximeter	Yes/No	Yes/No	_______
Central vein puncture set	Yes/No	Yes/No	_______
Chest decompression set	Yes/No	Yes/No	_______
Burn dressings	Yes/No	Yes/No	_______
Reflector lamp	Yes/No	Yes/No	_______
Protective helmet with forehead flashlight for each team member	Yes/No	Yes/No	_______
Protective reusable gloves	Yes/No	Yes/No	_______
Protective glasses	Yes/No	Yes/No	_______
Fixed radio station in the ambulance	Yes/No	Yes/No	_______
Handheld radio station	Yes/No	Yes/No	_______
Ultrasound	Yes/No	Yes/No	_______

5. **Do the stretchers in the ambulance have straps for securing the patient on the stretcher?**
  ○ No
  ○ Yes
  ○ Need (specify): _______

**EMERGENCY RESPONSE AND EFFECTIVENESS IN URGENT INTERVENTIONS, MASS CASUALTIES**

1. **Do you have a vehicle for mass casualties (prepared with a larger number of stretchers, medical supplies, and other equipment)?**
  ○ No
  ○ Yes
2. **Do you have a written plan/procedures that workers are familiar with in case of a mass casualty event?**
  ○ No
  ○ Yes
3. **Do you have triage tags? (in the car or bag)**
  ○ No
  ○ Yes
4. **If yes, do you use triage tags?**
  ○ No
  ○ Yes
5. **Have you had exercises for responding to mass casualties within your institution in the last 2 years?**
  ○ No
  ○ Yes
6. **If yes, how often have you had exercises in the last 2 years?**
  ○ Specify: ______________
7. **Have you had joint exercises with other rescue services (police, military, firefighters) in the last 2 years?**
  ○ No
  ○ Yes
8. **If yes, how often have you had such exercises in the last 2 years?**
  ○ Specify: ______________

**COMPLETED BY:**

- Name and surname: ______________
- Function: ______________
- Name and address of the healthcare institution: ______________
- Phone number: ______________
- Mobile phone number: ______________
- Email: ______________
- Municipality: ______________
- District: ______________
- Date: ______________

## Appendix B. Nomenclature (Terms and Abbreviations)

1. Hazards—potentially threatening events, as unusual occurrences or human activities, that may cause human casualties or injuries, destruction of property, social and economic disruptions or environmental degradatio (International Strategy for Disaster Risk Reduction [171];
2. Disasters—a serious disruption of the functioning of a community or a society involving widespread, material, economic or environmental losses and impacts, which exceeds the abilityof the affected community or society to cope using its own resources [171];
3. Disaster risk—the potential disaster losses, in lives, health status, livelihoods, assets and services, which could occur to a particular community or a society over some specified future time period [171];
4. Early warning system—the set of capacities needed to generate and disseminate timely and meaningful warning information to enable individuals, communities and organizations threatened by a hazard to prepare and to act appropriately and in sufficient time to reduce the possibility of harm or loss [171];
5. Emergency management—the organization and management of resources and responsibilities for addressing all aspects of emergencies, in particular preparedness, response and initial recovery steps [171];
6. Mitigation—the lessening or limitation of the adverse impacts of hazards and related disasters [171];
7. Natural hazard—natural process or phenomenon that may cause loss of life, injury or other health impacts, property damage, loss of livelihoods and services, social and economic disruption, or environmental damage [171];
8. Preparedness—the knowledge and capacities developed by governments, professional response and recovery organizations, communities and individuals to effectively anticipate, respond to, and recover from, the impacts of likely, imminent or current hazard events or conditions [171];
9. Recovery—the restoration, and improvement where appropriate, of facilities, livelihoods and living conditions of disaster-affected communities, including efforts to reduce disaster risk factors [171];
10. Resilience—the ability of a system, community or society exposed to hazards to resist, absorb, accommodate to and recover from the effects of a hazard in a timely and efficient manner, including through the preservation and restoration of its essential basic structures and functions [171];
11. Response—the provision of emergency services and public assistance during or immediately after a disaster in order to save lives, reduce health impacts, ensure public safety and meet the basic subsistence needs of the people affected [171];
12. Risk—the combination of the probability of an event and its negative consequences [171];
13. Technological hazard—A hazard originating from technological or industrial conditions, including accidents, dangerous procedures, infrastructure failures or specific human activities, that may cause loss of life, injury, illness or other health impacts, property damage, loss of livelihoods and services, social and economic disruption, or environmental damage [171];
14. Vulnerability—the characteristics and circumstances of a community, system or asset that make it susceptible to the damaging effects of a hazard [171];
15. Emergency Medical Services (EMS)—the organized medical response systems that provide care during emergencies;
16. Emergency Medical Response Systems (EMRS)—a broader system that encompasses various emergency medical services;
17. Mass casualty preparedness—the readiness and ability of EMS systems to handle incidents involving multiple casualties simultaneously;
18. Shift work—a scheduling method where EMS staff work rotating shifts to provide 24-h emergency coverage;
19. Pre-hospital care—medical care provided before the patient reaches a hospital, typically by EMS teams at the emergency site or during transport;
20. Pearson’s correlation—a statistical method used to measure the strength of a relationship between two variables;
21. Chi-square test—a statistical test used to determine if there is a significant association between categorical variables;
22. R^2^—coefficient of determination, indicating how well the regression model fits the data;
23. Β—beta coefficient, representing the standardized regression coefficient in the multivariate regression analysis;
24. *p*-value—statistical significance level, used to determine whether the results are statistically significant.
